# Hybrid Platinum(IV)-Naproxen Nanostructured Drugs Reprogram Melanoma Cells and Overpower Cisplatin

**DOI:** 10.3390/nano15171320

**Published:** 2025-08-28

**Authors:** Teodora Komazec, Dijana Bovan, Goran N. Kaluđerović, Ekatarina Mihajlović, Ivana Predarska, Duško Dunđerović, Evamarie Hey-Hawkins, Sanja Mijatović, Danijela Maksimović-Ivanić

**Affiliations:** 1 Department of Immunology, Institute for Biological Research “Siniša Stanković”—National Institute of the Republic of Serbia, University of Belgrade, Bulevar Despota Stefana 142, 11108 Belgrade, Serbia; teodora.komazec@ibiss.bg.ac.rs (T.K.); dijana.draca@ibiss.bg.ac.rs (D.B.); ekatarina.mihajlovic@ibiss.bg.ac.rs (E.M.); sanjamama@ibiss.bg.ac.rs (S.M.); 2 Department of Engineering and Natural Sciences, University of Applied Sciences Merseburg, Eberhard-Leibnitz-Strasse 2, 06217 Merseburg, Germany; goran.kaluderovic@hs-merseburg.de (G.N.K.); ivana_p90@hotmail.com (I.P.); 3 Institute of Bioanalytical Chemistry, Centre for Biotechnology and Biomedicine, Faculty of Chemistry, Leipzig University, 04109 Leipzig, Germany; hey@uni-leipzig.de; 4 Institute of Pathology, School of Medicine, University of Belgrade, Dr Subotića 8, 11000 Belgrade, Serbia; dusko.dundjerovic@med.bg.ac.rs; 5 Faculty of Chemistry and Chemical Engineering, Department of Chemistry, Babeș-Bolyai University, Str. Arany Janos Nr. 11, 400028 Cluj-Napoca, Romania

**Keywords:** anti-inflammatory drugs, drug delivery, melanoma, cisplatin conjugates, SBA-15

## Abstract

The concept of hybrid drugs that integrate cytotoxic and anti-inflammatory activity, enabling the simultaneous delivery of a chemotherapeutic agent and a non-steroidal anti-inflammatory drug (NSAID) into the tumor microenvironment (TME), was created with the aim of blocking the mitogenic signals that lead to tumor renewal. Here, we provide for the first time a detailed insight into the mechanism of action of a platinum(IV) complex based on the cisplatin (CP) scaffold bearing two deprotonated NSAID ligands (naproxenate (Npx)) in axial position ([CP(Npx)_2_]), free and immobilized in nanostructured silica SBA-15 (SBA-15|[CP(Npx)_2_]), in a melanoma model. The conjugate in free or loaded form diminished the viability of cancer cells more potently than CP, with an exceptional preference for the malignant phenotype. Type I and II programed cell death, senescence, and terminal differentiation of the surviving cell fraction were the basic mechanisms of action by which the new hybrid molecule achieved its effect in vitro. In the mouse melanoma model, the application of the therapeutic agents led to a reduction in tumor volume, extinguishing of intratumoral inflammation, and an overall better toxicity profile compared to CP. Overall, this approach improved the efficacy of chemotherapy by removing obstacles that cause chronic inflammation in the TME.

## 1. Introduction

Melanoma is one of the most commonly diagnosed cancers in adolescents and young adults [1,2] and is one of the most aggressive and deadly types of skin cancer [3,4]. Treatment decisions for melanoma are influenced by multiple factors, including the stage of disease, the presence of specific genetic mutations, the location of primary and metastatic lesions, and patient-related factors such as comorbidities and overall performance status. In addition to surgical treatment of early-stage melanoma, the standard of care for melanoma today consists of immune checkpoint inhibitors (e.g., pembrolizumab, nivolumab, and ipilimumab) and BRAF/MEK inhibitors [5]. Radiotherapy and chemotherapy (dacarbazine and temozolomide) have largely been replaced by immunotherapy and targeted therapies.

Cisplatin (CP) is one of the most successful and widely used anticancer drugs, which primarily leads to the death of tumor cells through interaction with DNA, but also affects healthy cells [6,7,8,9]. Platinum-based drugs are rarely used alone in melanoma, but rather as part of multi-drug regimens such as CP + vinblastine + temozolomide [10] or platinum + immunotherapy combinations. Chemo-immunotherapy approaches combining carboplatin/paclitaxel with immune checkpoint inhibitors have shown a synergistic effect in melanoma [11]. However, the main disadvantage of platinum(II) compounds, which limits their clinical use, is the lack of selectivity, i.e., toxicity, and the acquisition of resistance. To overcome these shortcomings, new platinum-based drugs with more suitable pharmacological properties have been developed. Indeed, it has been shown that platinum(IV) complexes, CP conjugates to which selected ligands are bound in the axial position, exhibit lower toxicity and higher selectivity. Platinum(IV) compounds have been shown to have anti-cancer properties [12]. They are designed as prodrugs, as the release of the biologically active ligands and the conversion of platinum(IV) into its active form, platinum(II), can occur upon reduction with various biomolecules (e.g., glutathione) [13,14,15]. For some platinum(IV) complexes, no reduction is required, but these complexes themselves have exceptional antitumor activity [14,15].

Inflammation is involved in cancer growth and promotes all stages of cancerogenesis. Chronic inflammation, infection, or autoimmunity are associated with the occurrence of about 15–20% of all cancer cases [16]. The most meritorious enzymes involved in the inflammatory process are cyclooxygenase 1 and 2 (COX-1 and COX-2), which are responsible for the biosynthesis of various pro-inflammatory mediators, such as prostaglandins, prostacyclin, and thromboxane [17]. Previous studies have shown that the expression of COX-2 is increased in malignant melanoma cells [18,19,20,21,22]. It is known that upregulation of COX-2 leads to increased cell proliferation and invasiveness, inhibition of apoptosis, stimulation of angiogenesis, immunosuppression, and release of mutagens in tumors [22,23]. One of the most important products of these enzymes is prostaglandin E2 (PGE2), which is the most frequently observed prostaglandin in various human malignancies [24,25]. PGE2 is released in an autocrine and paracrine manner and can therefore affect neighboring cells, the components of the tumor microenvironment (TME), and the tumor cells themselves [26]. PGE2, which plays a crucial role in cancerogenesis by stimulating cancer invasion and progression [24,25,26], is also a mediator of regeneration [27]. It has been shown that dying cells release this molecule and thereby stimulate the regeneration of damaged tissue. In tumor tissue, PGE2 can play an important role in increasing tumor mass by stimulating the division of adjacent cells in response to apoptosis that occurs spontaneously or as a result of applied therapy [28,29]. Increased release of PGE2 has often been associated with poor prognosis [18,24]. For these reasons, there is a need to remove PGE2-mediated mitogenic signals from the tumor environment. One of the basic approaches targeting the COX enzyme is the use of non-steroidal anti-inflammatory drugs (NSAIDs) [30]. In this way, they would potentially reduce the spread of tumor tissue by inhibiting mitogenic stimuli as well as cell proliferation, angiogenesis, and mutagenesis. In recent years, it has become increasingly clear that the widely used NSAID naproxen has considerable antitumor potential [31,32] and could be part of a future therapy.

**Figure 1:**
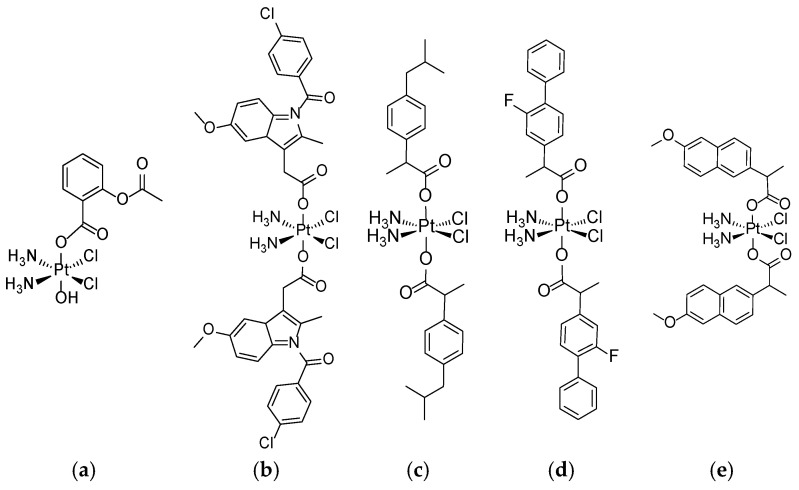
Selected cisplatin conjugates with various NSAIDs (employed as deprotonated carboxylic acids): (**a**) aspirin, (**b**) indomethacin, (**c**) ibuprofen, (**d**) flurbiprofen, and (**e**) naproxen. Complexes (**c**–**e**) are diastereomers.

So far, numerous hybrid molecules of CP and various NSAIDs as axial ligands (typically employed as deprotonated carboxylic acids) have been synthesized (Figure 1), e.g., aspirin [33], indomethacin [34,35], ibuprofen [34], flurbiprofen [36], ketoprofen, naproxen [37], etodolac, carprofen, sulindac [38], and many others. The positive role of chemotherapeutic agents in inducing tumor cell death would be combined with the suppression of the potentially mitogenic signal of PGE2. A significant antitumor effect has been demonstrated for a large number of them both in vitro and in vivo [39].

An additional advantage in reducing systemic toxicity, increasing selectivity, and targeting tumor tissue is achieved by using nanostructured silica materials (MSN), namely Santa Barbara Amorphous-15 (SBA-15) [40]. A major advantage of MSNs over other drug vehicles is their extremely low toxic profile in vivo [41]. SBA-15 enables the passive delivery of drugs into the tumor tissue due to the “enhanced permeability and retention effect”(EPR effect). At the same time, SBA-15 prevents hydrolysis, non-specific protein–drug interactions, ensures tailored drug release, and reduces undesirable side effects associated with many conventional drugs [42]. SBA-15 nanostructures loaded with platinum(IV) conjugates, [CP(NSAID)2], have shown potent antiproliferative activity against various breast cancer cell lines [43]. SBA-15 particles have also been used as carriers for platinum(IV) complexes with an oxaliplatin (Oxa) core carrying various anti-inflammatory drugs, [Oxa(NSAID)_2_] [44]. To date, various nanomaterials have been used for the delivery of platinum(IV) complexes, such as carbon-based materials, silicon-based materials, nanogold, polysiloxane liposomes, polymeric micelles, inorganic nanomaterials, and many others [45,46]. For example, Linming Li et al. synthesized naproxen platinum(IV) complex nanomaterials encapsulated in bovine serum albumin and demonstrated their antitumor activity [47].

The efficacy of platinum(IV) complexes with NSAIDs as ligands in the axial position has been investigated primarily in tumor types that are highly associated with inflammation. Among them are mammary tumors with very prominent inflammatory processes and colorectal cancer as a prototypical type of inflammation-related tumor [48].

Herein, we present for the first time in vitro and in vivo data obtained in a mouse melanoma model. A detailed insight into the cytotoxic potential and mode of action of the platinum(IV) naproxenate conjugate [CP(Npx)_2_] and the corresponding nanomaterial is provided, focusing not only on their superior cytotoxic properties, but also on their potential to induce senescence/differentiation in melanoma cells. The fact that an experimental hybrid molecule can trigger reprogramming of melanoma cells, leading to loss of malignant properties and eradication of protumor signals in the microenvironment, is one of the advances that this concept can offer in tumor therapy. In this way, advanced tumor forms can be converted into less aggressive ones, making them more sensitive to conventional treatment methods. Furthermore, the use of these and similar molecules, in combination with nanotechnology and for the purpose of targeted drug delivery, could significantly attenuate the adverse effects of cytostatic therapies.

## 2. Materials and Methods

### 2.1. Reagents

Dimethyl sulfoxide (DMSO), phosphate-buffered saline (PBS), acridine orange (AO), propidium iodide (PI), carboxyfluorescein diacetate succinimidyl ester (CFSE), ribonuclease A (RNase), fluoromount-G medium, trisaminomethane hydrochloride (TRIS HCl), 3-methyl adenine (3-MA), dithiothreitol (DTT) and glycerol, Folin and Ciocalteu’s phenol, 3,4-dihydroxy-L-phenylalanine (L-DOPA), naproxen, and CP were obtained from Sigma-Aldrich (St. Louis, MO, USA). Crystal violet (CV) was purchased from Merck Group (Darmstadt, Germany). Fetal bovine serum (FBS), cell culture medium Roswell Park Memorial Institute (RPMI) 1640 medium, RPMI 1640 medium without phenol red, Dulbecco’s Modified Eagle Medium (DMEM) high glucose, trypsin, and ethylenediaminetetraacetic acid (EDTA) were bought from Capricorn Scientific GmbH (Ebsdorfergrund, Germany). Paraformaldehyde (PFA), acrylamide/Bis solution 29:1 and *N*,*N*,*N*′,*N*′-tetramethylethylenediamine (TEMED) were purchased from Serva (Heidelberg, Germany). BioMount Aqua was obtained from BioGnost (Zagreb, Croatia). Fluorescein di-β-D-galactopyranoside (FDG), anti-Notch1 and anti-beta actin antibodies were acquired from Abcam (Cambridge, UK), while β-catenin and Oct-3/4 antibodies were obtained from Santa Cruz Biotechnology (Dallas, TX, USA). The penicillin/streptomycin solution was acquired from Biological Industries (Cromwell, CT, USA). Annexin V-FITC (AnnV) was bought from BD Pharmingen (San Diego, CA, USA), while ApoStat was from R&D Systems (Minneapolis, MN, USA). The trypan blye and triton X-100 were acquired from Fluka (Buchs, Switzerland). Oil red O solution and Mayer’s hematoxylin were obtained from BioOptica (Milan, Italy). Dihydrorhodamine 123 (DHR), 4-amino-5-methylamino-2′,7′-difluorofluorescein diacetate (DAF-FM diacetate), ammonium persulfate and PageRuler ladder, goat anti-mouse IgG (H+L), and gat anti-rabbit IgG (H+L) secondary antibodies were purchased from Thermo Fisher Scientific (Waltham, MA, USA). Sodium hydroxide (NaOH) was obtained from Lach-Ner (Neserratovice, Czechia). 3-(4,5-Dimethythiazol-2-yl)-2,5-diphenyltetrazolium bromide (MTT) and bovine serum albumin (BSA) were from AppliChem (St. Louis, MO, USA), while sodium dodecyl sulfate (SDS) and Tween 20 were ordered from AppliChem (Darmstadt, Germany). Acetic acid, methanol, and ethanol were purchased from Zorka (Šabac, Srbija). Electrochemiluminescence (ECL) was acquired from GE Healthcare (Buckinghamshire, UK), and the polyvinylidene difluoride (PVDF) membrane was purchased from Roche Diagnostics GmbH (Mannheim, Germany).

The physicochemical characterization of the [CP(Npx)_2_] conjugate, as well as nanostructures used in this study, including SEM analysis and nitrogen adsorption–desorption measurements, were previously reported [43]. Briefly, the BET surface area of SBA-15 was 517 m^2^ g^−1^, and upon loading with [CP(Npx)_2_], it decreased to 274 m^2^ g^−1^, indicating the successful incorporation of the complex into the mesopores. The particle sizes determined with SEM were ca. 200–400 × 600–800 nm. These materials were used in the present study without further modification.

### 2.2. Cell Culture and In Vitro Studies

The mouse cell lines B16 (melanoma), CT26 and MC38 (both colorectal carcinoma), NIH/3T3 (mouse fibroblast cell line), and the human melanoma (518A2, A-375, and FemX) were provided from ATCC (Rockville, VA, USA). The 4T1 cell line (mouse breast cancer cells) was donated by Prof. Nebojša Arsenijević from the Faculty of Medical Sciences, University of Kragujevac, Serbia.

The above-mentioned cell lines B16, 4T1, and CT26, and human melanomas were propagated in HEPES-buffered RPMI-1640 medium, while DMEM was used for the MC38 and NIH/3T3 cell lines. The medium was supplemented with 0.01% sodium pyruvate, 2 mM L-glutamine, 10% heat-inactivated FBS, as well as penicillin (100 units/mL) and streptomycin (100 μg/mL). All cells were grown in T-25 flasks in an incubator at 37 °C in a humidified atmosphere with 5% CO_2_.

To determine the viability of B16, 4T1, CT26, MC38, NIH/3T3, A-375, 518A2, and FemX cells, the following cell densities were used: 3 × 10^3^, 2 × 10^3^, 6 × 10^3^, 1 × 10^3^, 10^4^, 4 × 10^3^, 10^3^, and 5 × 10^3^ cells/well, respectively, in 100 µL of culture medium, in a 96-well plate. For flow cytometry, biochemical assays and Western blot (WB) B16 cells were seeded at a density of 1 × 10^5^ cells/well in 1 mL of culture medium in a 6-well plate. For all microscopic staining, B16 cells were seeded at a density of 1.6 × 10^4^ cells/well in 4-well chamber slides.

Stocks of the free conjugate were prepared in DMSO at a concentration of 10 mM and stored at −20 °C before use. The stock of CP was prepared in DMSO at a concentration of 10 mM fresh before use. The final concentration of DMSO in the working solutions was less than 0.1%. The stocks of SBA-15|[CP(Npx)_2_] were prepared in PBS at a concentration of 2 mg/mL, always fresh, immediately before use.

### 2.3. Cell Viability Assays

The mentioned cells were seeded overnight and then treated with a wide range of concentrations of the hybrid molecule [CP(Npx)_2_], CP as a positive control, or SBA-15|[CP(Npx)_2_] for 72 h. To compare the activity of the hybrid drugs with concomitant treatment with both subunits separately, B16 cells were treated simultaneously with CP and naproxen in the same ratio as for [CP(Npx)_2_] (namely 1:2) under the same conditions. To determine the potential selectivity of the compounds for the malignant phenotype, the NIH/3T3 cell line was treated with a range of concentrations starting from 10 µM for [CP(Npx)_2_], 100 µM for CP, and 100 µg/mL for SBA-15|[CP(Npx)_2_] material.

The MTT and CV assays were used to determine the potential effects of the treatment on cell viability. Following treatment, the medium was removed and the cells were washed with PBS, after which they were incubated with MTT solution (0.5 mg/mL) at 37 °C under standard conditions. After 30 to 60 min, when the formazan crystals formed, the solution was removed. The formazan crystals were then dissolved by adding DMSO.

For the CV assay, the treated cells were washed with PBS and fixed with 4% PFA for 15 min. Once the PFA was removed, the cells were dried for a few min. The 1% CV solution was added and the cells were incubated for 20 min at room temperature. Finally, the staining solution was removed and the cells were washed with tap water and dried. The crystals were then dissolved with 33% acetic acid.

Absorbance was measured at 540 nm, with the reference 670 nm for both assays using the SpectraMaxM5 multi-well plate reader (Molecular Devices, San Jose, CA, USA). Cell viability was calculated as a percentage of control (100%) representing untreated cells. Experiments were performed in triplicate. SigmaPlot 15.0 software and Microsoft Excel 2010 with a four-parameter logistic function were used to calculate IC_50_ values, which represent a 50% reduction in cell viability compared to untreated cells. GraphPad Prism 8.0.2 software (San Diego, CA, USA) with nonlinear regression analysis was used to generate dose–response curves.

### 2.4. Flow Cytometry

B16 cells were seeded in 6-well plates and allowed to adhere overnight before treatment with an IC_50_ dose of free and immobilized [CP(Npx)_2_]. After 72 h, the cells were washed with PBS, trypsinized, stained (except for CFSE and DHR, where cells were stained just before seeding), and analyzed by flow cytometry. All results were obtained using the CyFlow^®^ Space Partec (Partec GmbH, Münster, Germany) and the CytoFLEX flow cytometer (Beckman Coulter, Brea, CA, USA) and processed with FlowJo software v10 (Tree Star, Ashland, OR, USA).

#### 2.4.1. Detection of Cell Proliferation

The potential effect of [CP(Npx)_2_] and SBA-15|[CP(Npx)_2_] on cell proliferation was determined by staining cells with 1 μM CFSE (10 min incubation), which were then seeded and treated a day later. The cells were analyzed after 72 h.

#### 2.4.2. Detection of Apoptosis and Caspase Activation

To detect possible apoptosis, the cells were double-stained with AnnV (1.35 μg/mL) and PI (15 μg/mL). After a 15 min light-protected incubation at room temperature, the cells were resuspended in AnnV binding buffer and analyzed. On the other hand, the pan-caspase inhibitor ApoStat (0.5 μg/mL) was used to detect potential total caspase activation. The cells were incubated with ApoStat for 30 min at 37 °C, resuspended in PBS and analyzed.

#### 2.4.3. Detection of Autophagy

Cells were stained with 10 μM AO for 15 min at 37 °C, washed, resuspended in PBS, and analyzed to detect the possible occurrence of autophagy. To determine whether autophagy has a protective role or represents a form of treatment-induced cell death, the cells were exposed to the autophagy inhibitor 3-MA (1 mM) in concomitant treatment with the experimental conjugates. To confirm this assumption, an MTT viability test was performed.

#### 2.4.4. Detection of β-Galactosidase

To detect β-galactosidase activation, cells were incubated for 1 min at 37 °C with β-galactosidase substrate (fluorescein-di-β-D-galactopyranoside) at a concentration of 1 mM. The samples were analyzed within a short period of time.

#### 2.4.5. Measurement of Intracellular Reactive Oxygen and Nitrogen Species

To detect intracellular reactive oxygen species, cells were incubated for 20 min at 37 °C in the presence of 1 μM DHR 123 dye, seeded, and treated the next day. The cells were analyzed after 72 h. For detection of intracellular NO content, the treated cells were incubated for 1 h with 5 μM DAF-FM in medium without phenol red at 37 °C. In addition, the cells were incubated for 15 min at 37 °C in a medium without phenol red and FBS and then analyzed.

### 2.5. Microscopy

The melanoma cells were cultured in 4-well chamber slides. The next day, cells were treated with IC_50_ or MC_50_ doses of [CP(Npx)_2_] or SBA-15|[CP(Npx)_2_], respectively, for 72 h, then washed with PBS and fixed with PFA 4% for 15 min. The cells were washed again with PBS and then stained with the appropriate dye. The staining results were analyzed using a Zeiss Axio Observer Z1 fluorescence microscope (Carl Zeiss AG, Oberkochen, Germany) at 400× magnification.

#### 2.5.1. PI Staining

The PI solution consists of 50 μg/mL PI, Triton X-100 (0.1%), EDTA pH 8.0 (0.1 mM), and 85 μg/mL RNase in PBS. Cells were incubated with the PI dye solution for 1 min in the dark. The cells were washed and mounted with Fluoromount-G medium. At the end, a coverslip was placed over the mounting medium, and the chamber slides were analyzed. The nuclear surface area was quantified using ImageJ software (NIH image program, version 1.54).

#### 2.5.2. HE Staining

The cells were incubated with hematoxylin for 1–2 min and then washed several times with tap water. Thereafter, eosin was added to the cells and also washed with tap water after 30 s. BioMount Aqua was used as the embedding medium. The coverslips were mounted on chamber slides. Nuclear surface area and cell size were quantified using ImageJ software.

#### 2.5.3. Oil Red O Staining

The fixed cells were stained with the dye Oil red O for 20 min and then rinsed 5 times with tap water. Hematoxylin was then added and 1–2 min later the cells were washed with tap water. In addition, the cells were covered with BioMount Aqua and a coverslip was placed over them. The presence of lipid droplets was quantified using ImageJ software.

### 2.6. Tyrosinase Activity and Presence of Melanin

After 72 h of treatment, the melanoma cells were counted and 1 × 10^6^ cells/sample were used for both assays, while the results were compared with the same number of untreated cells. The cells were centrifuged at 2000 rpm for 5 min. For the detection of tyrosinase activity, the cell pellet was mixed with PBS pH 6.8–1% Triton X-100 and centrifuged at 10,000 rpm for 5 min. The supernatant was incubated with 2 mg/mL L-DOPA for 20 min at 37 °C, and after formation of a brown color, the absorbance of the samples was measured at 540 nm. To determine the presence of melanin, 1M NaOH was added to the cell pellet and incubated for 1 h at 60 °C. The absorbance of the supernatant was measured at 492 nm.

### 2.7. Western Blot Analysis

Previously seeded cells were treated with the IC_50_ or MC_50_ concentration of [CP(Npx)_2_] or the corresponding SBA-15 nanostructured material SBA-15|[CP(Npx)_2_] for 24 and 48 h. After these periods, the cells were lysed with protein lysis buffer and their concentration was determined using the Lowry protein assay. The cell lysates (30 μg) were separated by molecular weight on a 12% SDS–polyacrylamide gel together with a PageRuler ladder. The proteins from the gel were then transferred to the PVDF membrane by semi-dry transfer using the Fastblot B43 (BioRad, Göttingen, Germany) transfer system. The membrane was blocked with 5% BSA in 0.1% Tween 20/PBS. After 1 h, the membrane was washed with 0.1% Tween 20 in PBS and incubated overnight at 4 °C with the corresponding primary antibody (Notch 1, β-catenin, Oct-3/4, or β-actin). The next day, after repeated washing, appropriate secondary antibodies (goat anti-mouse IgG and goat anti-rabbit IgG) were applied to the membrane and incubated for 1 h, followed by the detection of the bands using the iBright™FL1500 Imaging System (Thermo Fisher Scientific, Waltham, MA, USA). The results were processed by iBright Analysis 5.2.1 Software (Thermo Fisher Scientific, Waltham, MA, USA).

### 2.8. Animals and In Vivo Studies

The C57BL/6 mice used for the in vivo experiments were obtained from the animal facility of the Institute for Biological Research “Siniša Stanković” (IBISS)—National Institute of the Republic of Serbia, University of Belgrade. The 6- to 8-week-old male and female animals used were kept under standard laboratory conditions (ad libitum food and water intake, and free of specific pathogens). The protocols used in the in vivo study and the proper manipulation of the animals were in accordance with the provisions of the European Community guidelines (86/609/EEC of 24 November 1986). Likewise, the handling of mice was performed in compliance with the provisions of the local Institutional Animal Care and Use Committee (IACUC). The final confirmation for in vivo studies was issued by the Veterinary Directorate, of the Ministry of Agriculture, Forestry, and Water Management of the Republic of Serbia (Permit No. 323-07-07906/2022-05).

To determine the potential therapeutic effect of the conjugates in vivo, B16 melanoma cells (1.8 × 10^5^ cells per mouse/in 100 μL PBS) were inoculated subcutaneously into the right dorsal flank of C57BL/6 mice. Treatment was initiated at the moment the tumors became palpable (7–10 days after inoculation). Treatments were administered by i.p. injections every other day, followed by a two-day break. The animals were divided in 4 groups: a group treated with [CP(Npx)_2_] (10 mg/kg in 4% DMSO/PBS), a group treated with SBA-15|[CP(Npx)_2_] (30 mg/kg in PBS), a group receiving 2 mg/kg CP in 4% DMF/PBS (positive control), and control animals receiving the solvent (4% DMSO or 4% DMF in PBS). The number of mice per group was approximately 10. The tumor size was measured twice a week and the volume was calculated using the formula length × width^2^ × 0.52 (mm^3^). During the experiment, changes in the animals’ body weight, biochemical urinary parameters, and behavioral changes were observed. The biochemical and hematological parameters in the urine were analyzed with Multistix 10 SG (Bayer; Leverkusen, Germany). Approximately on the 20th day after cell inoculation, the mice were euthanized, tumors were extracted and measured, and finally collected, together with the liver and kidneys for histological analysis.

For investigation of tumorigenic potential, B16 cells were treated in vitro with the IC_50_ or MC_50_ dose of [CP(Npx)_2_] or SBA-15|[CP(Npx)_2_], respectively, and after 72 h 1 × 10^5^ cells per mouse/in 100 μL, PBS was inoculated into C57BL/6 mice in the same manner as described above. The control was inoculated with untreated cells. Tumor size was monitored and measured throughout the experiment. The experiment was terminated when the tumor volume reached approximately 10% of the animal’s total weight.

### 2.9. Histopathological Analysis

The tumors, livers, and kidneys of the sacrificed animals underwent macroscopic examination before being immersed in 10% neutral buffered formalin (NBF) at a 10:1 fixative-to-tissue ratio for 24 h. The tissues were then sectioned along the largest tissue plane and further fixed for an additional 24 h. Tissue processing was carried out using an automated tissue processor (LOGOS One, Milestone Srl, Valbrembo, BG, Italy), followed by paraffin embedding using an embedding console (Tissue-Tek ^®^ TEC™ 5, Sakura, Torrance, CA, USA).

Tissue sections 4 µm-thick were obtained using a microtome (RM 2245, Leica Biosystems, Wetzlar, Germany) and mounted on glass slides. The sections were heated at 60 °C for 15 min, deparaffinized in xylene, and rehydrated through decreasing ethanol concentrations. Staining was performed using an automated slide stainer (MYREVA SS-30H, Myr, Tarragona, Spain). The sections were stained with hematoxylin (5 min), rinsed, differentiated in acid alcohol, and blued in tap water or blueing reagent. They were then counterstained with eosin (2 min), dehydrated in ethanol, cleared in xylene, and coverslipped.

Histopathological analysis was conducted using an Olympus BX43 microscope (Olympus Europa Holding GmbH, Hamburg, Germany).

### 2.10. Immunohistochemistry

Formalin-fixed, paraffin-embedded tissue sections (4 µm) were deparaffinized in xylene and rehydrated through a graded ethanol series. Antigen retrieval was performed using citrate buffer (pH 6.0) in a pressure cooker at 120 °C for 3 min. Endogenous peroxidase activity was quenched by incubation in 3% hydrogen peroxide for 10 min at room temperature. The following primary antibodies were employed: anti-HMGB1 (rabbit monoclonal antibody, clone EPR3507, and dilution 1:250; Abcam, Cambridge, UK), and anti-COX-2 (rabbit polyclonal antibody, catalog number AB15191, and dilution 1:100; Abcam, Cambridge, UK). All antibodies were applied according to the manufacturers’ instructions. Immunoreactivity was visualized using the EnVision™+ System-HRP (Dako, Agilent Technologies, Santa Clara, CA, USA), with diaminobenzidine (DAB) as the chromogen. Slides were counterstained with Mayer’s hematoxylin, dehydrated, cleared, and mounted with coverslips. Negative controls were prepared by omitting the primary antibody.

Slides were analyzed using an Olympus BX43 light microscope. For each antibody, two parameters were assessed: the percentage of positively stained cells (0–100%) and the staining intensity on a 3-tier scale (1 = weak, 2 = moderate, and 3 = strong). An immunoreactivity score (IRS) was calculated by multiplying these two values, yielding a total score range from 0 to 300 (IRS = intensity × percentage of positive cells). Mean values, standard deviations, and score ranges were subsequently calculated for each antibody.

### 2.11. Statistical Analysis

The data shown represent the mean ± standard deviation (SD) of at least three independent experiments, where a *p*-value of less than 0.5 was considered significant. Statistical significance between groups was determined using Student’s *t*-test and one-way analysis of variance (ANOVA) with Bonferroni correction. The non-parametric Mann–Whitney U test was used for the in vivo study. STATISTICA (data analysis software), version 8.0, was used for the statistical analysis of the data.

## 3. Results

### 3.1. Conjugate in Free and Nanoloaded Form Diminished the Viability of Cancer Cells In Vitro More Potently than Cisplatin

To determine the cytotoxic efficacy of [CP(Npx)_2_], four cell lines of mouse origin, including B16, MC38, 4T1, and CT26, as well as human A-375, 518A2, and FemX, were treated with various concentrations during a 72 h period. The cytotoxicity of [CP(Npx)_2_] and SBA-15|[CP(Npx)_2_] on the above mentioned cell lines, with CP as a reference drug, was evaluated by two different tests, MTT and CV. The activity of the experimental drugs was also compared to the activity of naproxen and CP in concomitant treatment against the same cell lines. The IC_50_ and MC_50_ values are given in Table 1.

Free [CP(Npx)_2_] shows effective antitumor activity in all tumor cell lines tested (Figures S1 and S2), with IC_50_ values below 3 μM. [CP(Npx)_2_] is significantly more effective than the parental CP, being at least 15 times more cytotoxic than this conventional chemotherapeutic agent. As expected, naproxen demonstrates no efficacy against the tested cell lines (>100 µM) (Figures S3 and S4). Of the cell lines tested, CT26 cells appear to be the least sensitive to [CP(Npx)_2_], which is consistent with their initial low sensitivity to CP. In general, advanced forms of colorectal cancer are resistant to CP, but not to other platinum-based drugs. In contrast, the other cell lines tested showed a good response to [CP(Npx)_2_] and achieved IC_50_ values in the nanomolar dose range. A mixture of CP and naproxen resulted in higher IC_50_ values in the same cell lines, indicating the advantages of the hybrid molecule concept compared to a multi-drug approach (Figures S3 and S4). Antitumor activity in the very low micromolar range was also observed for [CP(Npx)_2_], which is immobilized in SBA-15, against all cell lines except CT26 cells (Figure S1). As previously shown, SBA-15 itself does not affect cell viability [49].

Loading into SBA-15 did not improve the anticancer effect of [CP(Npx)_2_], with the exception of B16 cells regarding the CV assay (Table 1). An observed inconsistency between the viability assays used could be ascribed to the specific mode of action of the agents used in these cell lines. Indeed, the results of the MTT and CV assays can be influenced by alterations in mitochondrial respiration (in the case of MTT) and by changes in cell size, protein, or DNA content (in the case of CV).

Concurrently, the sensitivity of embryonic mouse fibroblasts NIH/3T3 to the same agents was also evaluated (Figure S5, Table 1) to determine the selectivity index (SI), which indicates the specificity of the compound towards cancer cells. The SI was calculated as the ratio of IC_50_ values for non-tumor cells vs. B16 cells. The cytotoxic potential of the free conjugate was approximately 10 times higher in cancer cells than in normal cells (SI = 13) and even higher in the case of SBA-15|[CP(Npx)_2_] with SI = 31 according to the MTT assay, indicating that the use of this mesoporous silica carrier significantly increases selectivity (Figure S5). Overall, an extraordinary preference of these agents for malignant phenotype was observed, while CP showed no selectivity in vitro (SI less than 1). Due to the great discrepancy between the antitumor activities of [CP(Npx)_2_] and CP, the B16 melanoma cell line was selected for further analysis.

### 3.2. Conjugate in Free and Nanostructured Form Downregulated Tumor Cell Viability in Pleiotropic Manner: From Programed Cell Death Type 1 and 2 to Inhibited Proliferation of Survived Cells

**Figure 2:**
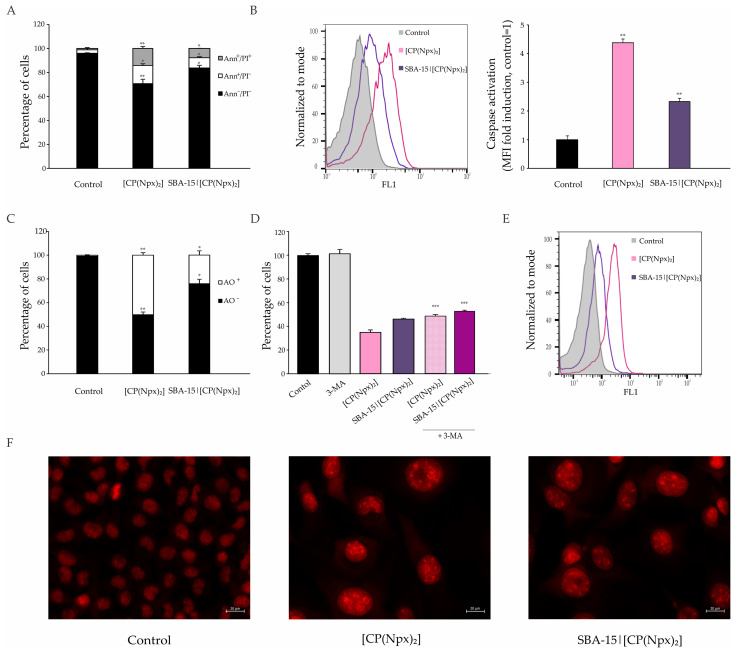
[CP(Npx)_2_] and SBA-15|[CP(Npx)_2_] induce caspase-dependent apoptosis together with autophagic cell death and loss of survived cell fraction dividing potential. The cells were treated with the IC_50_ of [CP(Npx)_2_] and the MC_50_ dose of SBA-15|[CP(Npx)_2_] for 72 h. Flow cytometric analysis of apoptosis by annexin V-FITC/propidium iodide (Ann/PI) double staining (**A**); total caspase activation after staining by Apostat (**B**); and autophagy after acridine orange staining (**C**) were completed. The effect of autophagy inhibition with 3-methyladenine (1 mM) on the viability of B16 cells (3-(4,5-Dimethythiazol-2-yl)-2,5-diphenyltetrazolium bromide, MTT assay) (**D**); flow cytometric analysis of cell proliferation by carboxyfluorescein diacetate succinimidyl ester (CFSE) staining (**E**); and fluorescence microscopy of PI-stained cells (**F**). Results are presented as mean values ± SD of three independent experiments while histograms are from one representative experiment. Statistical significance was determined and indicated as * *p* < 0.05, ** *p* < 0.01, and *** *p* < 0.001 compared to the untreated control.

Since the induction of apoptosis is nowadays a predominant concept in the tumor mass eradication, it was of interest to see whether [CP(Npx)_2_] initiates this type of cell death in the selected cell line. Therefore, Ann/PI double staining was performed after exposing the B16 cells to [CP(Npx)_2_], free and immobilized into SBA-15. The results presented in Figure 2A and Figure S6 indicate that both [CP(Npx)_2_] and SBA-15|[CP(Npx)_2_] led to an increase in the percentage of early and late apoptotic cells in B16 cultures compared to the untreated control, with the effect being more intense in the case of free [CP(Npx)_2_]. As a key parameter of apoptosis, caspase activity was further determined by flow cytometric analysis of Apostat-stained cells. The results presented in Figure 2B show that caspases were activated in both treatments, although the effect was stronger in cells treated with free [CP(Npx)_2_], suggesting the involvement of caspases in the realization of apoptosis.

Since autophagy usually acts as a self-protective and often counteracts treatment-induced apoptosis, the presence of autophagosomes in the cytoplasm of treated cells was assessed using AO staining. The level of cellular autophagic activity was increased in the treated cultures, as shown in Figure 2C and Figure S7, especially in the cells exposed to free [CP(Npx)_2_], where approximately 50% of the cell population exhibited increased autophagosome accumulation in the cytosol. To distinguish whether the observed autophagy was merely an attempt at survival or actually a form of cell death, the autophagy inhibitor 3-MA was administered at the same time and cell viability was estimated. The suppression of autophagy by 3-MA is based on blocking autophagosome formation by inhibiting class III phosphatidylinositol 3-kinases. As shown in Figure 2D, simultaneous treatment with [CP(Npx)_2_] and the specific autophagy inhibitor resulted in restoration of cell viability, indicating a destructive role of autophagy in both treatments—free and SBA-loaded [CP(Npx)_2_].

CFSE staining showed that the applied treatments affected cell division in a way that was opposite to the control culture (Figure 2E and Figure S8). Indeed, microscopic assessment of the cells that survived the treatment revealed a predominance of oversized nuclei (Figure S9A), in addition to a reduced amount of cells in the first place (Figure 2F). Altogether, it can be concluded that caspase-dependent apoptosis, together with autophagic cell death and loss of dividing potential of the survived cell fraction, are responsible for the viability reduction in the presence of both treatments.

### 3.3. Conjugate in Free and Nanostructured Form Induced Senescence and Directed B16 Cells Toward Primary Melanocytes

**Figure 3:**
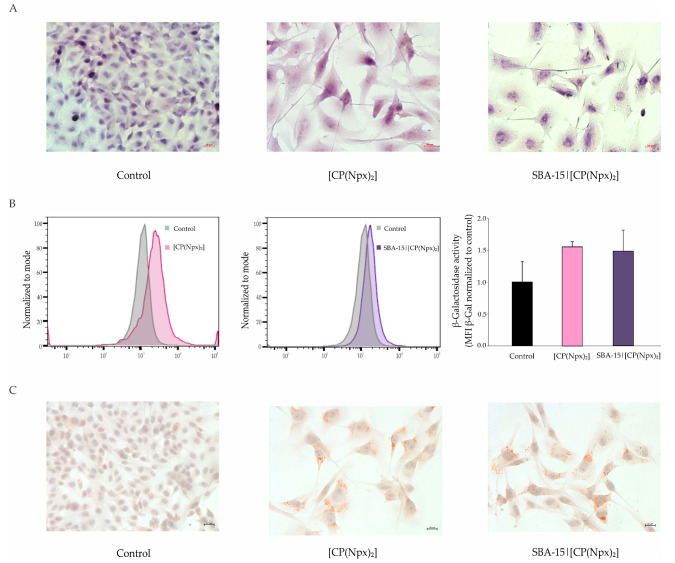
[CP(Npx)_2_] and SBA-15|[CP(Npx)_2_] induce senescence in B16 mouse melanoma cells. The cells were treated with the IC_50_ of [CP(Npx)_2_] and the MC_50_ dose of SBA-15|[CP(Npx)_2_] for 72 h and light microscopy of hematoxylin-eosin-stained B16 cells (**A**), and flow cytometric analysis of β-galactosidase activity after fluorescein di-β-D-galactopyranoside (FDG) staining (**B**) and light microscopy of Oil red O-stained B16 (**C**) were completed. Results show the mean values ± SD of three independent experiments, while the histograms and micrographs are from a representative one.

The inhibition of cell proliferation and the extremely enlarged nuclei of PI-stained cells observed upon treatment with free or SBA-15-loaded [CP(Npx)_2_] indicate changes in cellular phenotype, and thus, behavior. Conventional HE staining was performed to further characterize the cell shape and intracellular structures in more detail. Micrographs of HE-stained B16 cells showed that the applied treatments led to an appearance of the enlarged and flattened cells with enlightened cytoplasm (Figure 3B and Figure S9B), and together with introspections collected by PI staining about the nucleus oversize and the specific distribution of eu- and heterochromatin, it is suggested that survived cells may potentially be in a senescent state. Concordantly, a flow cytometric analysis of β-galactosidase activity, a main senescence biomarker, was next performed. Although in the case of SBA-15|[CP(Npx)_2_], β-galactosidase activity was less significantly increased than in cultures treated with the free hybrid molecule [CP(Npx)_2_], a significant increase in the expression of this enzyme was observed in both treatments compared to untreated cells (Figure 3B). As lipid droplets are abundant and enlarged in postmitotic and senescence-like cells, Oil red O staining was further performed in order to confirm the data obtained by flow cytometry. As evidenced in the light microscopy micrographs of cells exposed to treatment with free [CP(Npx)_2_] or, alternatively, SBA-15|[CP(Npx)_2_], numerous and significantly larger lipid droplets were found in the cytosol of treated cells compared to untreated cells (Figure 3C and Figure S9C). In summary, all features of survived cell fractions elaborated above underline the acquisition of a senescence-like phenotype in response to free and immobilized [CP(Npx)_2_].

**Figure 4:**
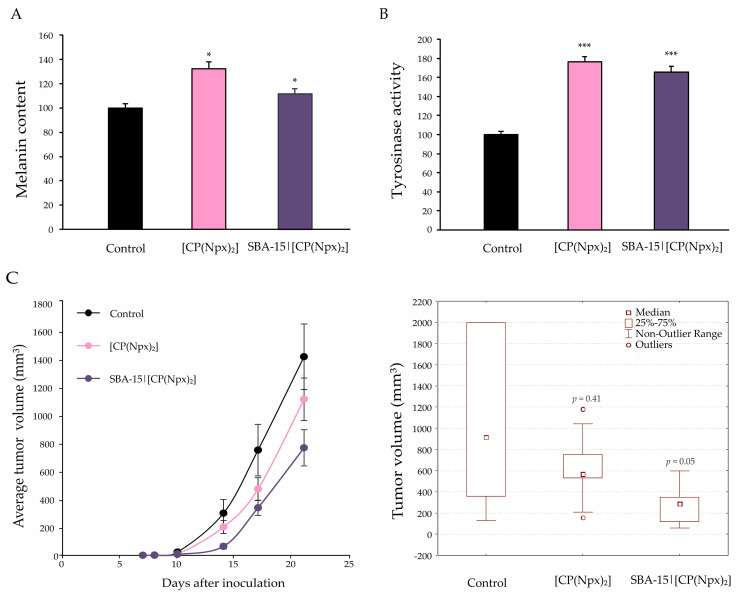
[CP(Npx)_2_] and SBA-15|[CP(Npx)_2_] induce differentiation of B16 cells into nonproliferative melanocytes. The cells were treated with the IC_50_ of [CP(Npx)_2_] and the MC_50_ dose of SBA-15|[CP(Npx)_2_] for 72 h, and melanin content (**A**), tyrosinase activity (**B**), and in vivo tumorigenicity tests (left—tumor growth curve, right—boxplot presentation of tumor volumes) (**C**) were completed (* *p* < 0.05 and *** *p* < 0.001 compared to the untreated control).

Aside from cellular senescence, microscopic evaluation of HE-stained cells after both treatments revealed the presence of one specific fraction of cells with a dendritic phenotype. A population of spindle-like cells with small nuclei and long tapering extensions was noticed in cultures exposed to [CP(Npx)_2_] or SBA-15|[CP(Npx)_2_]. Such morphological features indicated that the cells are likely transitioning to a more mature state through a process qualified as differentiation into melanocytes or transdifferentiation into Schwan-like cells. With the aim to define melanocytic differentiation, the signs of melanogenesis, as measured by tyrosinase expression and melanin synthesis, were further examined. Tyrosinase activity and, expectedly, content of melanin were significantly increased in B16 cells upon the treatment with [CP(Npx)_2_] or SBA-15|[CP(Npx)_2_], indicating terminal differentiation of melanoma cells towards the original melanocyte phenotype (Figure 4A,B). To check whether these cells possess the capability to develop tumors, B16 cells were cultured in the presence of [CP(Npx)_2_] or SBA-15|[CP(Npx)_2_] for 72 h and then inoculated into the right flank of C57BL/6 mice in vivo. Tumor formation was monitored continuously for 21 days. The growth curve is presented in Figure 4C (left). In comparison to the tumors derived from the untreated cells, the tumors formed from the cells cultured in the presence of [CP(Npx)_2_] or SBA-15|[CP(Npx)_2_] were smaller in size and volume, with more impressive values in the group of animals that received cells pretreated with SBA-15|[CP(Npx)_2_] (Figure 4C (right)). The data obtained indicate a significantly weaker tumorigenic potential of cells treated with SBA-15|[CP(Npx)_2_], not only compared to untreated cells, but also to B16 cells exposed to [CP(Npx)_2_]. This result supports the concept of using nanostructured technologies not only to improve drug delivery, but also to reprogram the phenotype, leading to a reduction in malignant potential.

### 3.4. [CP(Npx)_2_] Promoted Oxidative/Nitrosative Stress While Drug Differentially Regulate the Expression of Stemness Markers Depending on Formulation

**Figure 5:**
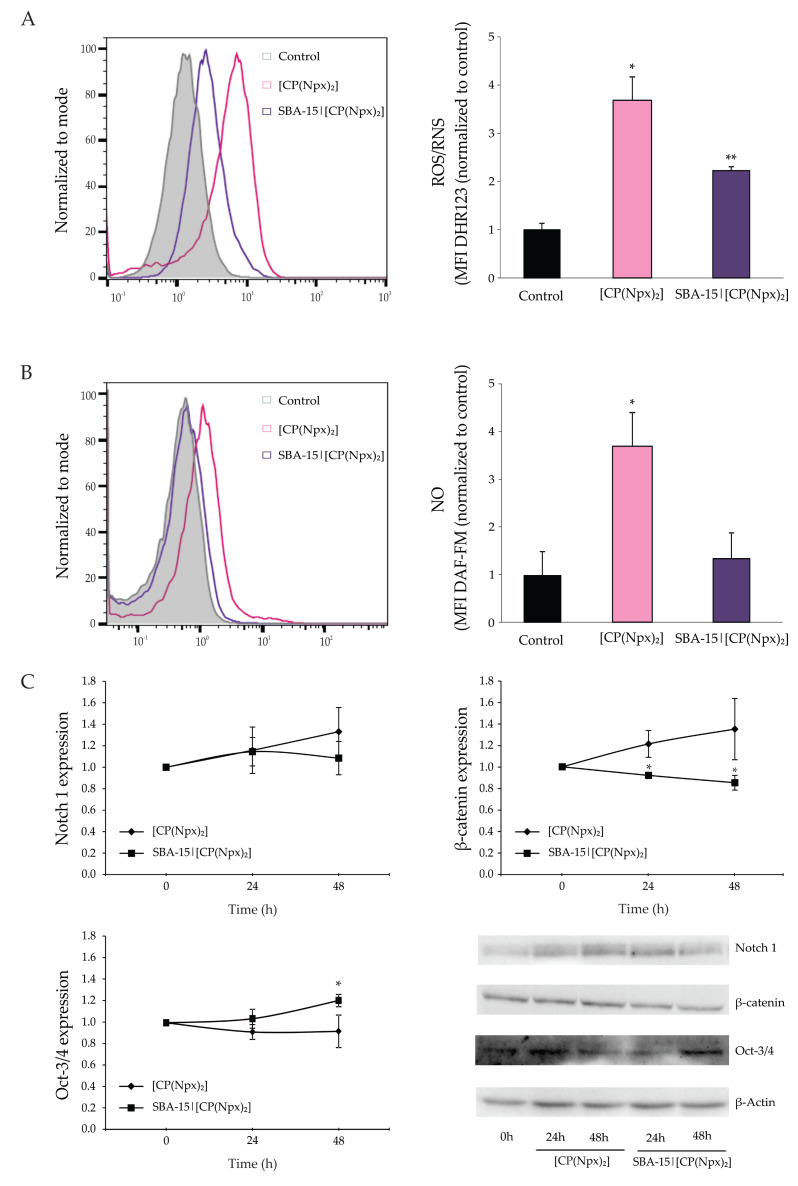
Effect of [CP(Npx)_2_] and SBA-15|[CP(Npx)_2_] on reactive oxygen/nitrogen species (ROS/RNS) production and expression of Notch 1, β-catenin, and Oct-3/4 proteins in B16 cells. The cells were treated with the IC_50_ of [CP(Npx)_2_] and the MC_50_ dose of SBA-15|[CP(Npx)_2_] for 72 h, and production of ROS/RNS by dihydrorhodamine 123 (DHR 123) (**A**) and intracellular NO by 4-Amino-5-methylamino-2′,7′-difluorofluorescein diacetate (DAF-FM) (**B**) were completed (left—representative of three independent experiments, right—mean values ± SD of three repeated experiments). The expression of Notch 1, β-catenin, and Oct-3/4 was evaluated during 48 h by Western blot analysis (**C**). Protein levels were expressed relative to β-actin. All data are presented as mean ± SD, * *p* < 0.05, and ** *p* < 0.01 compared to the untreated control.

The initiation of apoptosis is closely associated with reactive oxygen and nitrogen species (ROS/RNS) production in response to a certain type of antitumor agent with cytotoxic potential. To evaluate their contribution to the cytotoxicity of the applied treatments, endogenous NO and ROS/RNS were quantified with redox-sensitive dyes and subjected to flow cytometric analysis. The results show a dramatic increase in ROS levels after 72 h of cultivation in the presence of [CP(Npx)_2_] or SBA-15|[CP(Npx)_2_] compared to untreated cultures (Figure 5A), suggesting that ROS are likely involved in the induction of cell damage. In terms of NO content, a significant enhancement in fluorescence intensity was observed when [CP(Npx)_2_] was added to the cell culture compared to the control sample, but also to the cells treated with SBA-15|[CP(Npx)_2_] (Figure 5B). The fact that [CP(Npx)_2_] led to a much more intense oxidative burst and thus to more extensive apoptosis as well as autophagy compared to SBA-15|[CP(Npx)_2_] emphasizes the role of nanotechnology not only in drug delivery, but also in the dynamics of drug release within the cell. Changes in the dynamics of drug release can significantly influence the anti-tumor effect of the drug used.

To determine how treatment with free or SBA-15-loaded [CP(Npx)_2_] affects the signaling pathways that determine stem cell choice and differentiation status, the expressions of Notch 1, β-catenin, and Oct-3/4 were examined at the indicated time points. The expression of Notch 1 and β-catenin was stably upregulated by [CP(Npx)_2_], while Oct-3/4 remained unchanged. In contrast, SBA-15|[CP(Npx)_2_] induced downregulation of β-catenin, but stable positive regulation of Oct-3/4, while Notch 1 returned to basal levels after a slight upregulation (Figure 5C).

### 3.5. Immobilized Conjugate Was Superior in Melanoma Growth Reduction than Free Compound

**Figure 6:**
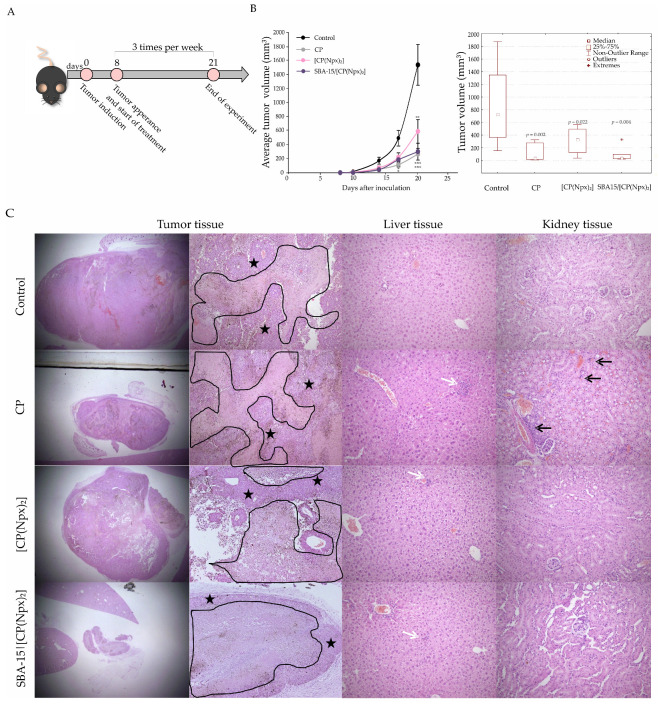
[CP(Npx)_2_] and SBA-15|[CP(Npx)_2_] efficiently reduced melanoma growth in vivo. The animals were treated with 10 mg/kg [CP(Npx)_2_], 30 mg/kg SBA-15|[CP(Npx)_2_], or 2 mg/kg CP. Schematic presentation of in vivo experiment (**A**); tumor growth curve and boxplot presentation of tumor volumes (* *p* < 0.05, ** *p* < 0.01, and *** *p* < 0.001) (**B**). Representative micrographs of the most significant histopathological alterations of tumor tissues (12.5× and 40× magnification), liver, and kidney tissues (200× magnification) (**C**). Tumor tissue necrosis is encircled with a black line, and viable tissue is marked with black stars. White arrows point to oligocellular necrosis, and black arrows denote foci of inflammation.

To further detect the antitumor capacity of both prodrugs—free and immobilized [CP(Npx)_2_] into SBA-15, a syngeneic mouse model of melanoma was employed. Tumors were induced by subcutaneous inoculation of B16 cells into the right flank of C57BL/6 mice, and treatment has started on day 8, when a first tumor became noted (Figure 6A). CP, as a positive control, was applied in a therapeutic dose, while the conjugates were given at a dose of 10 and 30 mg/kg three times a week, enforcing the same administration regimen as CP. Because of its proven inefficacy in tumor growth reduction and also its lack of toxicity, SBA-15 was excluded from the experiment [49]. The results obtained reveal that all three treatments significantly diminished tumor growth (Figure 6B). Nevertheless, SBA-15|[CP(Npx)_2_] was more efficient than [CP(Npx)_2_], which is what was expected due to the EPR effect as the basis of nanostructured particle-mediated drug delivery [41].

It is well known that nerve damage, nephrotoxicity, and hepatotoxicity are the key factors restraining the usage of platinum-based drugs. The animals receiving CP showed atypical behavioral characteristics such as anxiety, increased irritability, piloerection, and whimpering compared to the other three groups, while body weight was not significantly altered in all experimental groups (Figure S10). Furthermore, a histopathological examination of the tumor tissue, liver, and kidney by HE staining was performed (Figure 6C). Morphologically, all tumors exhibit an identical pattern—they are composed of epithelioid cells with the presence of fine dark pigment, noticeable geographic areas of necrosis, multiple foci of apoptonecrosis, and pronounced vascularization. The extent of tumor necrosis was lowest in the control group, while the highest values were observed in the conjugate-treated groups. Additionally, mitotic activity, expressed as the number of mitoses per 2 mm^2^, was also highest in the control group (57), while the mitotic rate was decreased in all treated groups ([CP(Npx)_2_]: 35, SBA-15|[CP(Npx)_2_]: 33, CP: 38.8), showing decreased proliferative nature of the tumor tissue.

The liver tissue of all groups, including the control group, maintained its lobular architecture, with low mitotic activity, without signs of hepatotoxicity except for some rare oligocellular necrosis. No significant morphological differences were observed between the groups. As far as renal tissue is concerned, rare and discrete morphological changes were observed almost exclusively in the group of animals receiving CP in the form of focal fibrosis, rare tubular dilatation, and minimal inflammatory foci, indicating possible nephrotoxicity. In contrast, the kidneys of the animals that received free or immobilized conjugate have an unaltered architecture with a clearly distinguishable cortex and medulla and are free of any pathological features that could impair renal function. In addition, evaluation of biochemical and hematological parameters in the animals’ urine revealed no difference between treated and untreated animals, suggesting that the physiological status of the animals is unchanged in the monitoring period (Table S1).

Overall, these results suggest an advantage of using platinum(IV) complexes over the commercially available chemotherapeutic CP. The dual release probably contributes synergistically to the observed therapeutic effect and emphasizes the potential of CP conjugates as multifunctional anticancer agents. Nevertheless, further studies on organ toxicity and with larger sample sizes are required.

### 3.6. Both Free and Immobilized Drug Conjugate Suppressed Inflammation in Tumor Tissue

**Figure 7:**
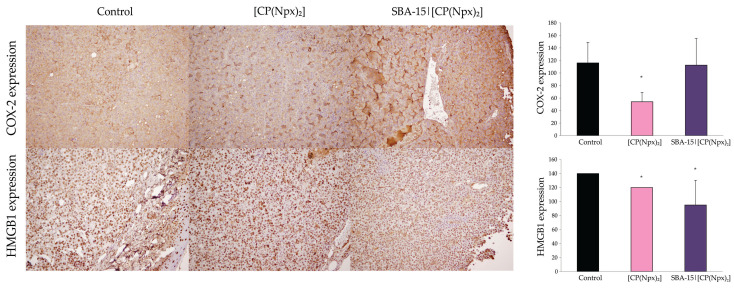
[CP(Npx)_2_] and SBA-15|[CP(Npx)_2_] suppressed inflammation inside tumor tissue. The animals were treated with a dose of 10 mg/kg [CP(Npx)_2_] and 30 mg/kg SBA-15|[CP(Npx)_2_]. COX-2 and HMGB1 expressions were evaluated by light microscopy (200× magnification). Representative micrographs of tumor sections for each group are presented, while the results are expressed as mean ± SD (n = 5) and * *p* < 0.05 compared to the untreated control.

In order to examine the effect of the new compounds on the parameters of inflammation in the TME, the animals were treated as described above. At the end of the treatment, the animals were sacrificed, the tumors were isolated, and the expression of COX-2 and HMGB1 was analyzed by immunohistochemistry. Compared to untreated animals, COX-2 expression was significantly reduced in animals receiving [CP(Npx)_2_] (Figure 7). On the other hand, no statistically significant reduction was observed in animals treated with the nanoformulation, probably due to a slower release and thus a lower but sustained concentration of the drug. Furthermore, the expression of the HMGB1 protein decreased significantly in both treatments, indicating that the extinction of the inflammatory process in the TME proves the efficacy of the applied therapy.

## 4. Discussion

Since its clinical introduction in the late 1970s, CP remained a cornerstone of cytotoxic chemotherapy. However, its widespread use is limited by significant off-target toxicity, affecting both malignant and healthy cells, as well as the rapid development of chemotherapy resistance [9]. The search for novel cytostatic agents with improved efficacy and selectivity toward tumor cells while minimizing adverse effects on normal tissue has long been a central driving force in global oncology research. As clinical practice demonstrated that treatment with a single cytostatic drug usually leads to the development of resistance, therapy protocols involving the simultaneous administration of several drugs with distinct mechanisms of action are increasingly being used [50]. This approach is particularly relevant in light of emerging insights into the TME, including its architecture, multicellular organization, and capacity for self-maintenance. Multi-targeting strategies aim to produce agents with higher selectivity for tissues undergoing malignant transformation, often combining direct cytotoxic potential against rapidly dividing cells with the targeting of some specific tumor-promoting features of the TME. One of the hallmark characteristics of the TME is a state of chronic inflammation that continuously drives its expansion [16]. Thus, the use of NSAIDs as an antitumor strategy has recently become more popular due to the growing evidence of inflammation-driven tumor progression and its contribution to the failure of conventional therapies [16]. The multiple challenges led to the development of the concept of hybrid molecules which combine a cytotoxic and an anti-inflammatory subunit and allow the simultaneous delivery of a metal-based agent and NSAIDs into the TME. Here, we provide a detailed insight into the mechanism of action of a platinum(IV) complex based on the CP scaffold carrying the anti-inflammatory drug naproxen (as a deprotonated anionic ligand) immobilized in nanostructured silica SBA-15. The platinum(IV) complex carrying naproxenate in the axial position has already been used for in vitro testing on the breast cancer cell lines MCF-7, MDA-MB-231, and MDA-MB-435, the colon cancer cell lines HCT116, and CT26, and the lung cancer A549, but has never been tested on a melanoma model before [51,52,53]. Good antiproliferative performance in vitro was also observed in highly chemoresistant tumors, such as malignant pleural mesothelioma [37]. As presented in this study, [CP(Npx)_2_] and SBA-15|[CP(Npx)_2_] were very effective against the tested cell lines in vitro.

Higher cytotoxic activities in tumor cell lines than in normal cells, as measured by SI values, indicate that this complex is significantly less toxic to healthy fibroblasts NIH/3T3 in contrast to commercially available CP. Moreover, the cisplatin-based prodrug [CP(Npx)_2_] was more than four hundred times more effective compared to the parental CP (according to the MTT assay). A similar observation was made in a study by Ravera et al. [37]. In the cell lines of colon adenocarcinomas HT-29, HCT116, and SW480, lung carcinoma A549, ovarian endometroid adenocarcinoma A2780, and malignant pleural mesothelioma MSTO-211H, the platinum(IV) complex containing axial naproxen (2-(6-methoxynaphthalen-2-yl)propanoic acid) was several dozen times more active than CP and Oxa alone. The lower IC_50_ values of the prodrugs compared to CP can be attributed to their higher intracellular accumulation. Namely, platinum(IV) complexes have a high lipophilicity, which enables better passage through cell membranes, which in turn increases intracellular concentrations [34]. Ravera et al. explained the trend of the activity of platinum(IV) complexes containing one axial naproxen in terms of lipophilicity [37]. CP, as a parent molecule, obviously has a lower uptake capacity, which is confirmed by the fact that only 1% of CP reaches DNA, while the rest is bound to many biomolecules in the bloodstream [54]. It might be assumed that the cytotoxicity profile of platinum(IV) conjugates is primarily due to their good lipophilicity and high cellular accumulation.

Multi-drug therapy is often limited by the different pharmacokinetic profiles and molecular targets of the co-administered agents, which can lead to unpredictable therapeutic outcomes and variable efficacy. To address this issue, intracellular activation of platinum(IV) complexes is utilized as a platform that enables the delivery of multiple drugs in a single dose, thus circumventing the challenges of combination therapy. As proof of this concept, [CP(Npx)_2_], which was investigated in this study, showed significantly higher activity than its components applied separately in a 1:2 ratio. This discrepancy in cytotoxicity could be attributed to the biological activity of the platinum(II) core of [CP(Npx)_2_], highlighting its advantages.

[CP(Npx)_2_] exerted its cytotoxic effect against the melanoma cell line by inducing apoptosis, autophagy, and blocking the cell division of the survived population. The production of ROS and RNS, measured by DHR and DAF, was significantly increased in response to the applied treatments, indicating their involvement in drug-induced cytotoxicity. Programed cell death type 1 and 2 was more pronounced in cells exposed to [CP(Npx)_2_] than the SBA-15-loaded compound. Previously, apoptosis was shown to be in the background of the effect of a platinum(IV) complex containing one naproxen in the colon cancer cell line HCT 116 and the lung adenocarcinoma A549 cells [37]. Namely, in the same study, a downregulation of the anti-apoptotic BCL-2 and an upregulation of the pro-apoptotic BAD and BAX were found, indicating the realization of the mitochondrial pathway of apoptosis. Tolan et al. also described apoptosis as a response of breast tumor cells to the presence of a platinum(IV) complex monosubstituted with naproxen [51]. It is evident that the diverse biological, metabolic, and genetic backgrounds of tumor cell lines tailor the different mechanisms of action of the hybrid molecules, leading to similar efficacy.

Research over the last fifteen years has shown that CP and carboplatin can induce senescence in various cancer cell lines [55]. CP loaded into SBA-15 has also been shown to act as a prosenescent drug in B16F10 cells [56]. The presented study is probably a rare example of a platinum(IV) complex conjugated to an NSAID, [CP(Npx)_2_], which is capable of converting tumor cells from proliferating to a senescent/differentiated state. Namely, the viable cell fraction that survived the treatment exerted senescent characteristics, previously being arrested in growth and a flattened in their morphology with many enlarged lipid droplets intracellularly. Enlarged cell size after treatment explains the higher IC_50_ doses obtained with the CV assay compared to MTT, emphasizing the limited relevance of this assay as an indicator of cell viability. Finally, increased activity of lysosomal galactosidases, which serve as a main senescence marker, was observed in B16 cell cultures treated with [CP(Npx)_2_] and the SBA-15-loaded form. Senescence in melanoma cells is often accompanied by cellular differentiation, or alternatively, transdifferentiation [57]. Judging by a significant increase in both tyrosinase activity and melanin content, the treated B16 cells entered the terminal differentiation pathway to primary, non-proliferative melanocytes.

Once tumor cells have undergone terminal differentiation, their ability to proliferate further and form a progressive tumor is often permanently aborted. Indeed, B16 cells previously exposed to [CP(Npx)_2_] or SBA-15|[CP(Npx)_2_] for 72 h display remarkably lower potential for tumor formation in syngeneic strain, indicating not only their abrogated proliferative capacity, but also their diminished progenitor properties. In the background of this phenomenon is a signaling network with key players, such as β-catenin, Notch 1, and Oct-3/4, which regulate self-renewal, differentiation, and tumorigenicity, but also escape cytotoxic treatments [58]. Interestingly, Notch 1 and β-catenin were upregulated during 48 h incubation with [CP(Npx)_2_]. On the other hand, loading to nanostructured SBA-15 led to distinct regulation of the same signaling pathways, resulting in decreased β-catenin and significantly upregulated Oct-3/4. The antitumor activity of the compound correlated with a transient upregulation of Notch 1 and β-catenin, possibly reflecting an early compensatory or differentiation-associated response [59,60]. Considering the dual role of these pathways, their activation in this context may contribute to growth arrest or modulation of tumor cell plasticity rather than classical oncogenic signaling [61,62,63]. In the form of nanomaterial, the same conjugate affects the mentioned signaling platform differently. Although Oct-3/4 in this specific combination is classically associated with pluripotency and self-renewal [64], its upregulation in the context of reduced β-catenin activity and unaltered Notch 1 levels may shift the cellular fate towards senescence [62,65]. This could represent a “failed reprogramming” phenotype, in which incomplete or unbalanced dedifferentiation triggers stress responses and stable cell cycle arrest [66,67]. Such findings highlight the complexity of transcriptional reprogramming under therapeutic pressure and the potential for non-linear cellular responses to antitumor agents [59,68].

Among the first data on the in vivo efficacy of platinum(IV) complexes containing an NSAID are those of Cheng et al. [69]. They demonstrated that asplatin, a prodrug of CP carrying an acetylsalicylicate molecule in the axial position, has higher antitumor efficacy with lower toxicity compared to CP [69]. Concerning the CP prodrug with one naproxen moiety in the axial position, a mouse xenograft model bearing MDA-MB-231 tumor cells was assessed and a remarkable inhibition of tumor growth was accomplished [70]. In the present study, [CP(Npx)_2_] and SBA-15|[CP(Npx)_2_] were less efficient compared to CP in a syngeneic model of mouse melanoma induced in C57BL/6 mice, but still very effective in reducing tumor growth. While immobilization into SBA-15 did not improve the efficacy of the drug in vitro, SBA-15|[CP(Npx)_2_] was more efficient compared to free [CP(Npx)_2_] in vivo. The superior effect of the nanoformulation is based on the enhanced recruitment of nanoparticles into the tumor tissue regarding the abnormal architecture of intratumoral blood vessels and interstitial fluid retention. Although the EPR effect can contribute to the passive accumulation of nanoparticles in tumor tissue in vivo, the intracellular uptake of SBA-15|[CP(Npx)_2_] may occur through passive fluid-phase uptake and macropinocytosis, a non-specific, energy-dependent internalization pathway that is active in many cancer cells. Their potential to take up substances from extracellular spaces by macropinocytosis results from their increased metabolic activity and their strategy of intensive intercellular exchange. As we previously reported [71], SBA-15 nanostructures are effectively internalized by melanoma cells in this way. This selective uptake correlates with the reduced ability of differentiated, non-malignant cells to undergo macropinocytosis and could thus partly explain the observed improved selectivity. In addition, the cylindrical shape of SBA-15 stimulates macropinocytosis in cancer cells more potently than spheroidal nanoparticles [72].

Although studies on the behavior of the [CP(Npx)_2_] conjugate and SBA-15|[CP(Npx)_2_] under simulated intracellular or extracellular conditions has not been performed, our in vivo histopathological results provide clear evidence for the biological relevance of the proposed activation pathway. In particular, tumor tissue from the treated animals showed clear signs of an anti-inflammatory response, including decreased expression of the inflammatory mediators COX-2 and HMGB1 and an increase in necrotic areas. These findings are consistent with the expected pharmacological effect of naproxen [73] and support the hypothesis that the hybrid complex releases both CP and bioactive naproxen upon cellular uptake and intracellular reduction. Consequently, both active subunits, CP and naproxen, influence the cell viability of tumor cells and in parallel alter the TME by modulating inflammatory and mitogenic signals.

A great discrepancy, and thus a potential preference for the free and immobilized [CP(Npx)_2_] over the widely used CP, may exist in terms of toxicity. Although CP is associated with numerous toxicities, such as gastrotoxicity, myelosuppression, ototoxicity, and allergic reactions, its primary dose-limiting side effect is nephrotoxicity [74]. Indeed, in our study, visible but modest signs of nephrotoxicity were present on tissue cross-sections of CP-treated animals. These changes included focal fibrosis, occasional tubular dilatation, and minimal inflammatory foci, which is largely concordant with the literature data indicating renal tissue damage and raised inflammatory content [74]. The moderate nephrotoxicity in this study, in contrast to the known renal damage caused by this cytostatic drug, is due to the use of DMF as a cisplatin solvent. Indeed, DMF is known to attenuate the toxic effects of cisplatin on renal tissue through activation of the NRF2 signaling pathway and inhibition of NFkB [75]. Notwithstanding these data, histopathological analysis of the renal tissue from CP-treated animals indicated initial damage that has not yet manifested itself at the biochemical level. Obviously, platinum(IV) complexes have the potential to overcome the disadvantages of using platinum(II) drugs.

Judging by the improved selectivity for tumor tissue in vitro and the reduction in tumor growth observed in vivo, the use of SBA-15 as a silica carrier could be a very valuable drug delivery option. The study presented here also provides evidence that [CP(Npx)_2_] and SBA-15|[CP(Npx)_2_] induce senescence/differentiation in vitro in the treatment of mouse melanoma. The increasing interest of the scientific community today is focused on differentiation-based therapy as a non-aggressive approach to limit tumor growth, which consists of losing the malignant properties of the cancer cell. Senescence- and differentiation-inducing agents are being proposed as novel promising alternatives to conventional, non-selective cytotoxic chemotherapy.

It is well known that chronic inflammation, i.e., tumor-associated inflammation, creates a strong immunosuppressive environment. Shutting down tumor-promoting inflammatory pathways can also reprogram the TME and make it available for an effective immune response, thus increasing sensitivity to immunotherapy. Key target molecules in this network are actually the members of the COX-2 PGE2 signaling pathway. Accordingly, it was shown that the administration of NSAIDs, together with checkpoint inhibitors, potentiated the infiltration of CD8 T cells and synergized with PD1 blockade [76]. We propose that therapy with this type of hybrid drug can multiply the effectiveness of immunotherapy through both increased tumor immunogenicity and enhanced anti-tumor immune response. Such peculiarities consider the possibility of applying the hybrid molecules containing NSAID and cytostatic in the combined protocol with immunotherapy.

## 5. Conclusions

Current research in tumor biology increasingly points to the nature of tumor-promoting inflammation in the TME, which limits the effect of applied chemotherapeutic agents and leads to continuous tumor progression. The platinum(IV)-naproxen conjugate [CP(Npx)_2_] and the corresponding nanomaterial SBA-15|[CP(Npx)_2_] showed exceptional activity in vitro, favoring the malignant phenotype and overcoming CP potential in vitro. The idea of synchronized quenching of tumor-promoting inflammation with the induction of malignant cell death can be an approach that influences tumor progression and enhances the efficacy of chemotherapy in a pleotropic manner. The hybrid molecule, applied in naked and nanoloaded form, induced programed cell death type 1 and 2 of melanoma cells, while the surviving subpopulation became senescent and adopted the phenotype of mature, non-proliferative melanocytes that were previously blocked in their division. Consistently, [CP(Npx)_2_] and SBA-15|[CP(Npx)_2_] effectively reduced melanoma growth in C57BL/6 mice, with a more favorable toxicity profile than the clinically used chemotherapeutic agent CP. The background to this effect is a well-synchronized action between the anti-inflammatory and cytostatic subunit that not only acts on the bulk cells, but also interferes with the TME in multiple ways. Bearing in mind the multicellular nature and the complexity of the interactions in the tumor mass, this approach can make a significant contribution to overcoming the existing limitations and improving the effect of chemotherapeutic agents.

## Figures and Tables

**Table 1:** IC_50_ [µM] or MC_50_ [µg/mL] values of CP, naproxen, [CP(Npx)_2_], SBA-15|[CP(Npx)_2_], and CP + naproxen (1:2) determined with CV and MTT assays (72 h).

Cell Lines	Compound or Material						
		[CP(Npx)_2_]	SBA-15|[CP(Npx)_2_] *	CP	Naproxen	CP + Naproxen	SBA-15|[CP(Npx)_2_] **
B16	MTT	0.04 ± 0.01	0.08 ± 0.01	18.92 ± 0.29	>100	10.4 ± 0.8	0.61 ± 0.02
	CV	0.11 ± 0.01	0.07 ± 0.01	29.63 ± 2.14	>100	15.6 ± 0.5	0.58 ± 0.02
CT26	MTT	1.18 ± 0.10	3.96 ± 0.06	4.42 ± 0.21	>100	5.19 ± 0.22	34.875 ± 0.49
	CV	2.15 ± 0.21	5.61 ± 0.22	8.27 ± 0.14	>100	6.82 ± 0.2	48.87 ± 1.58
MC38	MTT	0.08 ± 0.01	0.27 ± 0.03	1.46 ± 0.12	>100	3.98 ± 0.38	2.35 ± 0.21
	CV	0.12 ± 0	0.49 ± 0.01	2.39 ± 0.14	>100	4.38 ± 0.41	4.31 ± 0.12
4T1	MTT	0.11 ± 0.01	0.44 ± 0.01	1.33 ± 0.02	>100	2.39 ± 0.07	3.85 ± 0.01
	CV	0.19 ± 0.01	0.07 ± 0.04	2.08 ± 0.03	>100	2.57 ± 0.21	6.09 ± 0.38
A-375	MTT	0.1 ± 0.01	0.3 ± 0.03	5.12 ± 0.43	>100	4.76 ± 0.23	2.67 ± 0.23
	CV	0.17 ± 0.0	0.47 ± 0.03	6.75 ± 0.59	>100	7.17 ± 0.44	4.08 ± 0.29
FemX	MTT	0.49 ± 0.05	0.7 ± 0.01	3.43 ± 0.26	>100	2.72 ± 0.01	6.09 ± 0.07
	CV	0.38 ± 0.03	1.93 ± 0.02	3.4 ± 0.3	>100	2.46 ± 0.15	16.02 ± 1.44
518A2	MTT	0.06 ± 0.0	0.13 ± 0.01	3.02 ± 0.15	>100	2.94 ± 0.25	1.13 ± 0.11
	CV	0.06 ± 0.0	0.13 ± 0.01	4.15 ± 0.35	>100	2.93 ± 0.06	1.15 ± 0.12
NIH/3T3	MTT	0.54 ± 0.03	/	4.67 ± 0.33	/	/	18.73 ± 1.68
	CV	0.98 ± 0.02	/	14.51 ± 0.22	/	/	31.05 ± 2.35

* recalculated based on EDX; ** in µg/mL; and /—not tested.

## Data Availability

Data supporting obtained results can be obtained from the authors upon request.

## Supplementary Materials

The following supporting information can be downloaded at: https://www.mdpi.com/article/10.3390/nano15171320/s1, Figure S1: Effect of [CP(Npx)_2_] and SBA-15|[CP(Npx)_2_] on viability of mouse cancer cells; Figure S2: Effect of [CP(Npx)_2_] and SBA-15|[CP(Npx)_2_] on viability of human cancer cells; Figure S3: Effect of cisplatin, naproxen and their mixture on viability of mouse cancer cells; Figure S4: Effect of cisplatin, naproxen and their mixture on viability of human cancer cells; Figure S5: Effect of [CP(Npx)_2_] and SBA-15|[CP(Npx)_2_] on viability of mouse NIH/3T3; Figure S6: Dot-plot representation of annexin V-FITC/PI staining after B16 cells treatment with [CP(Npx)_2_] and SBA-15|[CP(Npx)_2_]; Figure S7: Dot-plot representation of acridine orange staining after B16 cells treatment with [CP(Npx)_2_] and SBA-15|[CP(Npx)_2_]; Figure S8: Effect of [CP(Npx)_2_] and SBA-15|[CP(Npx)_2_] on proliferation potential of B16 cells; Figure S9: Effect of [CP(Npx)_2_] and SBA-15|[CP(Npx)_2_] on nuclear and cell size; Figure S10: Average mouse body weight; Table S1: Biochemical and hematological parameters of urine.

## Abbreviations

The following abbreviations are used in this manuscript:

NSAID	Nonsteroidal anti-inflammatory drug
TME	Tumor microenvironment
CP	Cisplatin
Npx	Naproxenate
SBA-15	Santa Barbara Amorphous-15, nanostructured silica
i.e.,	Id est, in other words
DNA	Deoxyribonucleic acid
e.g.,	For example
COX-1	Cyclooxygenase 1
COX-2	Cyclooxygenase 2
PGE2	Prostaglandin E2
MSN	Nanostructured silica materials
EPR effect	Enhanced permeability and retention effect
Oxa	Oxaliplatin
[CP(Npx)_2_]	Platinum(IV)-naproxenate conjugate
DMSO	Dimethyl sulfoxide
PBS	Phosphate-buffered saline
AO	Acridine orange
PI	Propidium iodide
CFSE	Carboxyfluorescein diacetate succinimidyl ester
RNase	Ribonuclease A
TRIS HCL	Trisaminomethane hydrochloride
3-MA	3-methyl adenine
DTT	Dithiothreitol
L-DOPA	3,4-dihydroxy-L-phenylalanine
CV	Crystal violet
FBS	Fetal bovine serum
RPMI	Roswell Park Memorial Institute, cell culture medium
DMEM	Dulbecco’s Modified Eagle Medium
EDTA	Ethylenediaminetetraacetic acid
PFA	Paraformaldehyde
TEMED	*N*,*N*,*N*′,*N*′-tetramethylethylenediamine
FDG	Fluorescein di-β-D-galactopyranoside
AnnV	Annexin V-FITC
DHR	Dihydrorhodamine 123
DAF-FM	4-amino-5-methylamino-2′,7′-difluorofluorescein
NaOH	Sodium hydroxide
MTT	3-(4,5-Dimethythiazol-2-yl)-2,5-diphenyltetrazolium bromide
BSA	Bovine serum albumin
SDS	Sodium dodecyl sulfate
ECL	Electrochemiluminescence
PVDF	Polyvinylidene difluoride
B16	Mouse melanoma cell line
CT26	Mouse colorectal carcinoma cell line
MC38	Mouse colon adenocarcinoma cell line
4T1	Mouse mammary carcinoma cell line
NIH/3T3	Mouse fibroblast cell line
CO_2_	Carbon dioxide
C57BL/6	Black laboratory mouse
IACUC	Institutional Animal Care and Use Committee
i.p.	Intraperitoneal injection
NBF	Neutral buffered formalin
SI	Selectivity index
