# Supplementary material for: Hybrid Platinum(IV)-Naproxen Nanostructured Drugs Reprogram Melanoma Cells and Overpower Cisplatin
Source: Nanomaterials (Basel). 2025 Aug 28;15(17):1320. doi: 10.3390/nano15171320 (PMC12430603; doi:10.3390/nano15171320)
Supplement: Supplementary file 1 [file nanomaterials-15-01320-s001.zip › nanomaterials-3759991-supplementary.pdf]

Supplementary file

Hybrid Platinum(IV)-Naproxen Nanostructured Drugs Reprogram Melanoma Cells and Overpower Cisplatin

Teodora Komazec [1], Dijana Bovan [1], Goran N. Kaluđerović [2], Ekatarina Mihajlović [1], Ivana Predarska [2,3], Duško Dunđerović [4], Evamarie Hey-Hawkins [3,5], Sanja Mijatović [1] and Danijela Maksimović-Ivanić [1,*]

[1] Department of Immunology, Institute for Biological Research "Siniša Stanković"—National Institute of the Republic of Serbia, University of Belgrade, Bulevar Despota Stefana 142, 11108 Belgrade, Serbia; teodora.komazec@ibiss.bg.ac.rs (T.K.); dijana.draca@ibiss.bg.ac.rs (D.B.); ekatarina.mihajlovic@ibiss.bg.ac.rs (E.M.); sanjamama@ibiss.bg.ac.rs (S.M.)

[2] Department of Engineering and Natural Sciences, University of Applied Sciences Merseburg, Eberhard-Leibnitz-Strasse 2, DE-06217 Merseburg, Germany; goran.kaluderovic@hs-merseburg.de (G.N.K.); ivana_p90@hotmail.com (I.P.)

[3] Institute of Bioanalytical Chemistry, Centre for Biotechnology and Biomedicine, Faculty of Chemistry, Leipzig University, 04109 Leipzig, Germany; hey@uni-leipzig.de

[4] Institute of Pathology, School of Medicine, University of Belgrade, Dr Subotića 8, 11000 Belgrade, Serbia; dusko.dundjerovic@med.bg.ac.rs

[5] Faculty of Chemistry and Chemical Engineering, Department of Chemistry, Babeș-Bolyai University, Str. Arany Janos Nr. 11, RO-400028 Cluj-Napoca, Romania; evamarie.hey@ubbcluj.ro

* Correspondence: nelamax@ibiss.bg.ac.rs

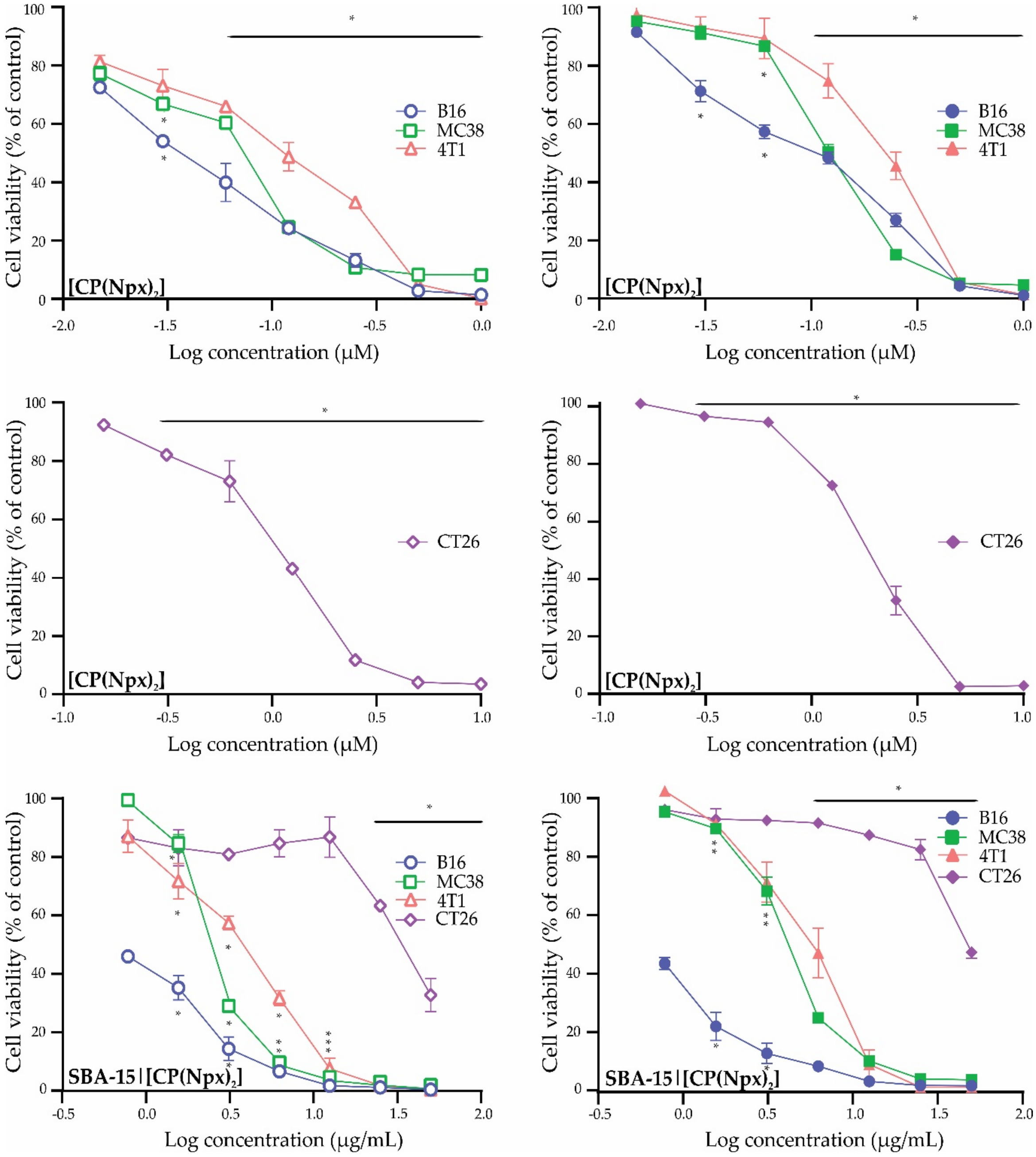

Figure S1: Effect of [CP(Npx)2] and SBA-15|[CP(Npx)2] on viability of mouse cancer cells. Cells were exposed to a wide range of concentrations of [CP(Npx)2] and SBA-15|[CP(Npx)2] for 72 h. Cell viability was determined using MTT (left) and CV (right) assays. Data are expressed as percentage of control ± SD from one of three independent experiments. * $p < 0.05$ compared to control.

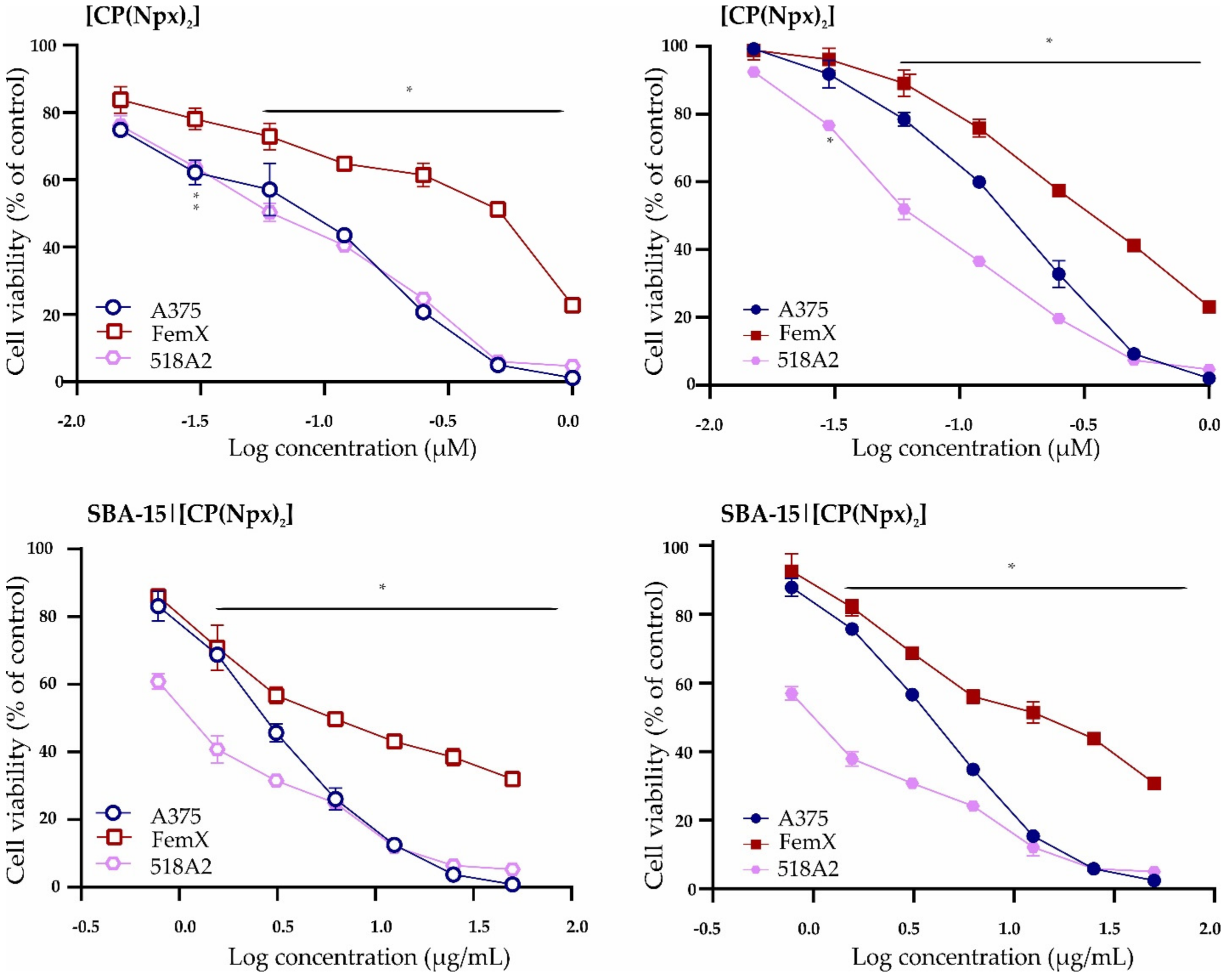

Figure S2: Effect of $[CP(Npx)_2]$ and SBA-15|$[CP(Npx)_2]$ on viability of mouse cancer cells. Cells were exposed to a wide range of concentrations of $[CP(Npx)_2]$ and SBA-15|$[CP(Npx)_2]$ for 72 h. Cell viability was determined using MTT (left) and CV (right) assays. Data are expressed as percentage of control ± SD from one of three independent experiments. * $p < 0.05$ compared to control.

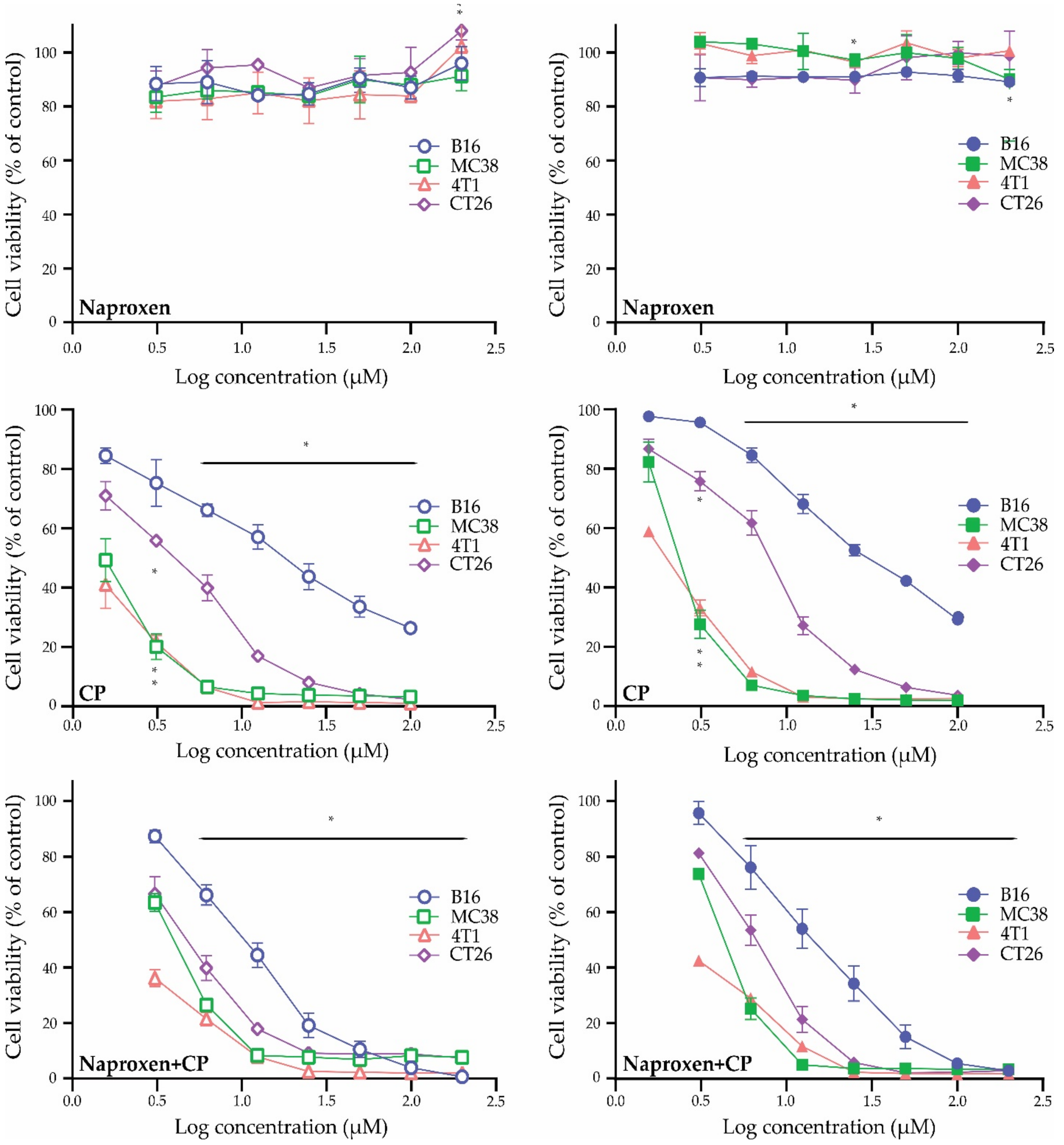

Figure S3: Effect of naproxen, CP and their mixture on viability of mouse cancer cells. Cells were exposed to a wide range of concentrations of CP, naproxen and their combination for 72 h. Cell viability was determined using MTT (left) and CV (right) assays. Data are expressed as percentage of control ± SD from one of three independent experiments. * $p < 0.05$ compared to control.

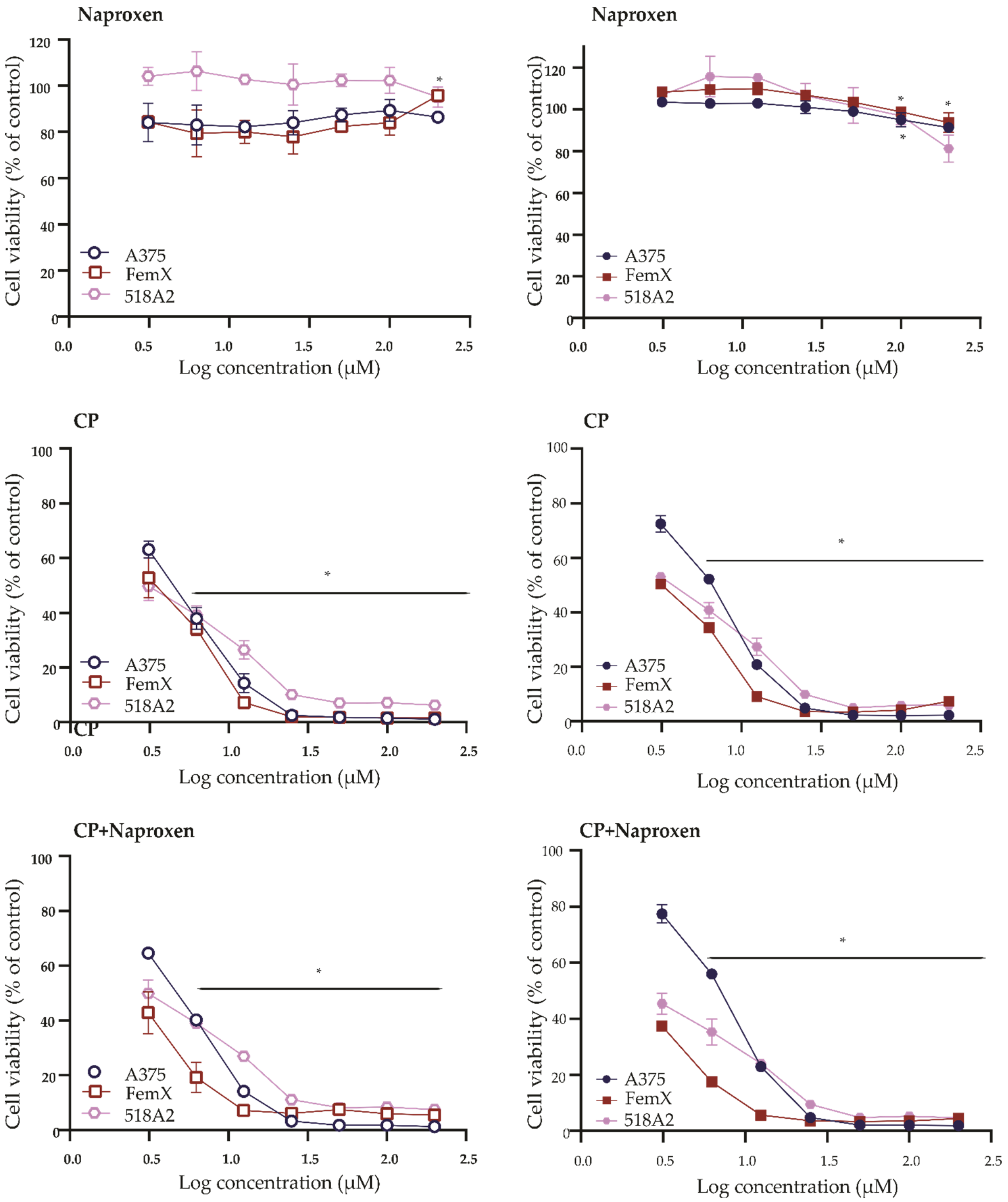

Figure S4: Effect of naproxen, CP and their mixture on viability of human cancer cells. Cells were exposed to a wide range of concentrations of CP, naproxen and their combination for 72 h.

Cell viability was determined using MTT (left) and CV (right) assays. Data are expressed as percentage of control ± SD from one of three independent experiments. * $p < 0.05$ compared to control.

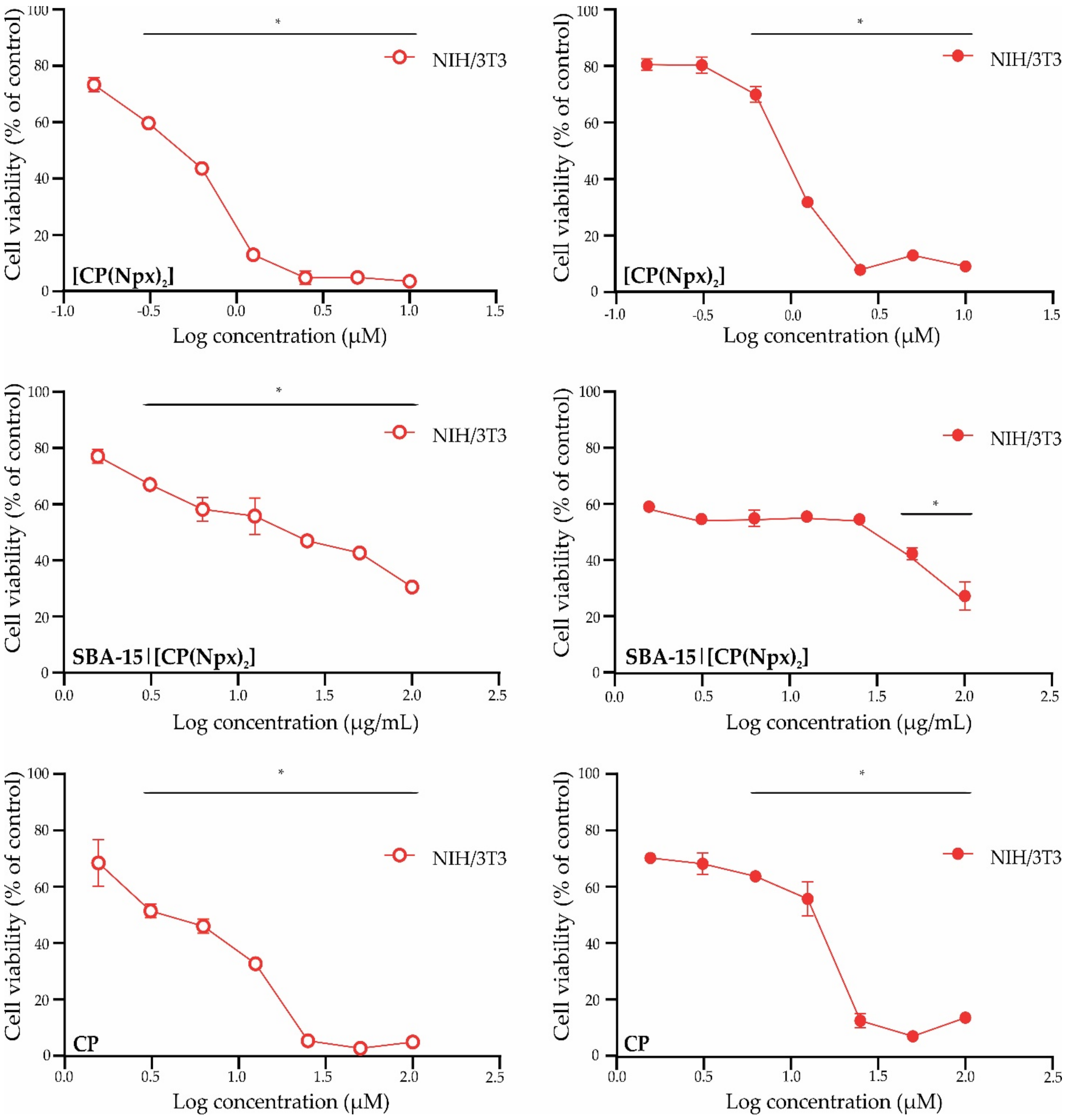

Figure S5: Effect of $[CP(Npx)_2]$ and SBA-15|$[CP(Npx)_2]$ on viability of mouse NIH/3T3. The NIH/3T3 cell line was exposed to a wide range of concentrations of $[CP(Npx)_2]$, SBA-15|$[CP(Npx)_2]$ and CP for 72 h. Cell viability was determined using MTT (left) and CV (right) assays. Data are expressed as percentage of control ± SD from one of three independent experiments. * $p < 0.05$ compared to control.

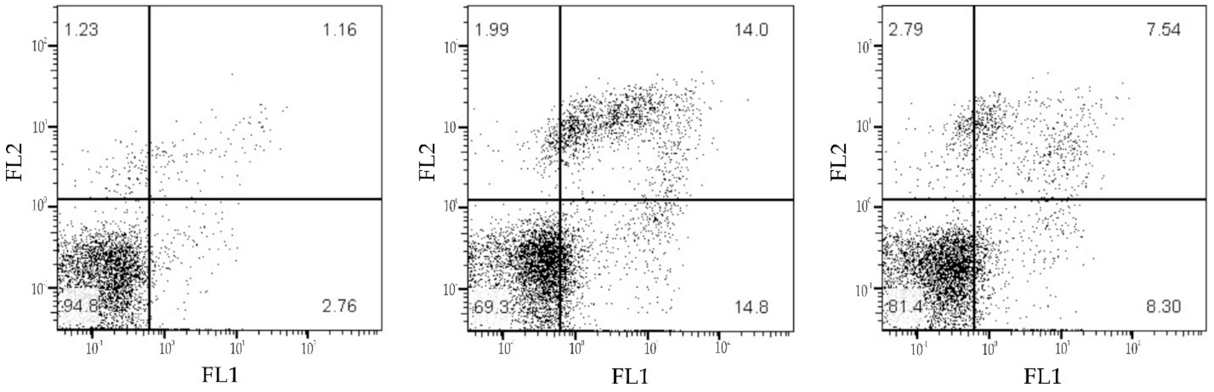

Control [CP(Npx)2] SBA-15|[CP(Npx)2]

Figure S6: Dot-plots show Annexin V-FITC/PI staining after treatment of B16 cells with [CP(Npx)2] and SBA-15|[CP(Npx)2]. Cells were treated with the IC_{50} of [CP(Npx)2] and the MC_{50} dose of SBA-15|[CP(Npx)2] for 72 h. One (representative) of three independently performed experiments is shown.

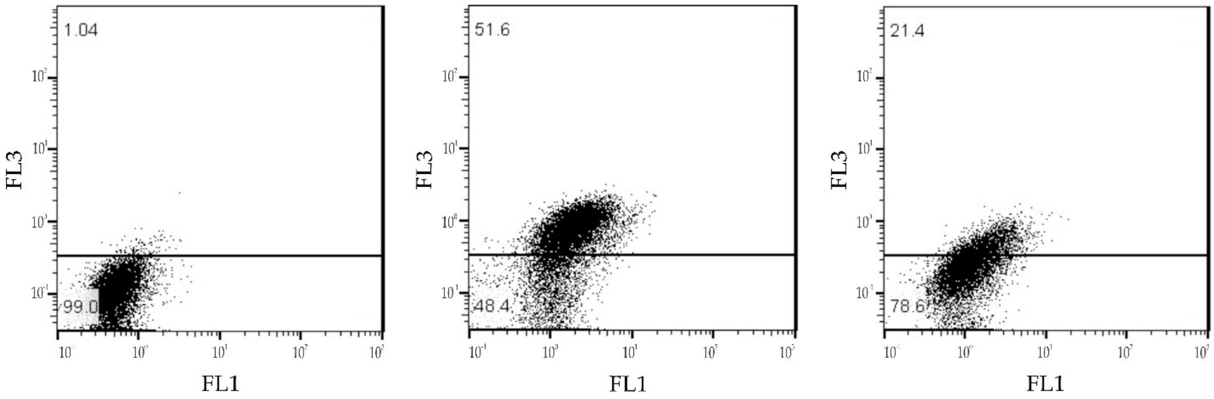

Control [CP(Npx)2] SBA-15|[CP(Npx)2]

Figure S7: Dot-plots show acridine orange staining after treatment of B16 cells with [CP(Npx)2] and SBA-15|[CP(Npx)2]. Cells were treated with the IC_{50} of [CP(Npx)2] and the MC_{50} dose of SBA-15|[CP(Npx)2] for 72 h. One (representative) of three independently performed experiments is shown.

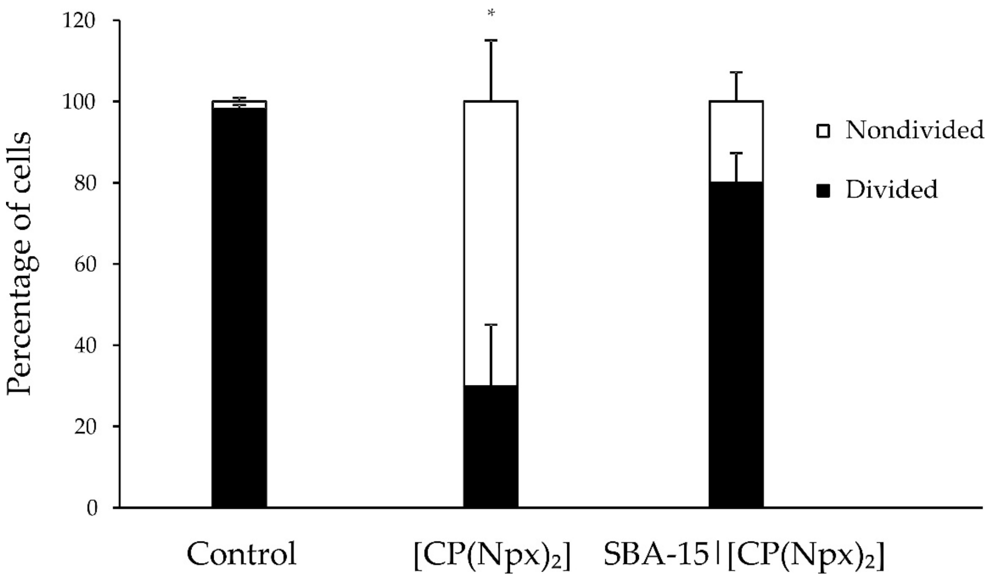

Figure S8: Effect of [CP(Npx)$_2$] and SBA-15|[CP(Npx)$_2$] on proliferation potential of B16 cells. Cells were treated with the IC_{50} of [CP(Npx)$_2$] and the MC_{50} dose of SBA-15|[CP(Npx)$_2$] for 72 h. Data from three independent experiments are expressed as mean ± SD. * $p < 0.05$ compared to control.

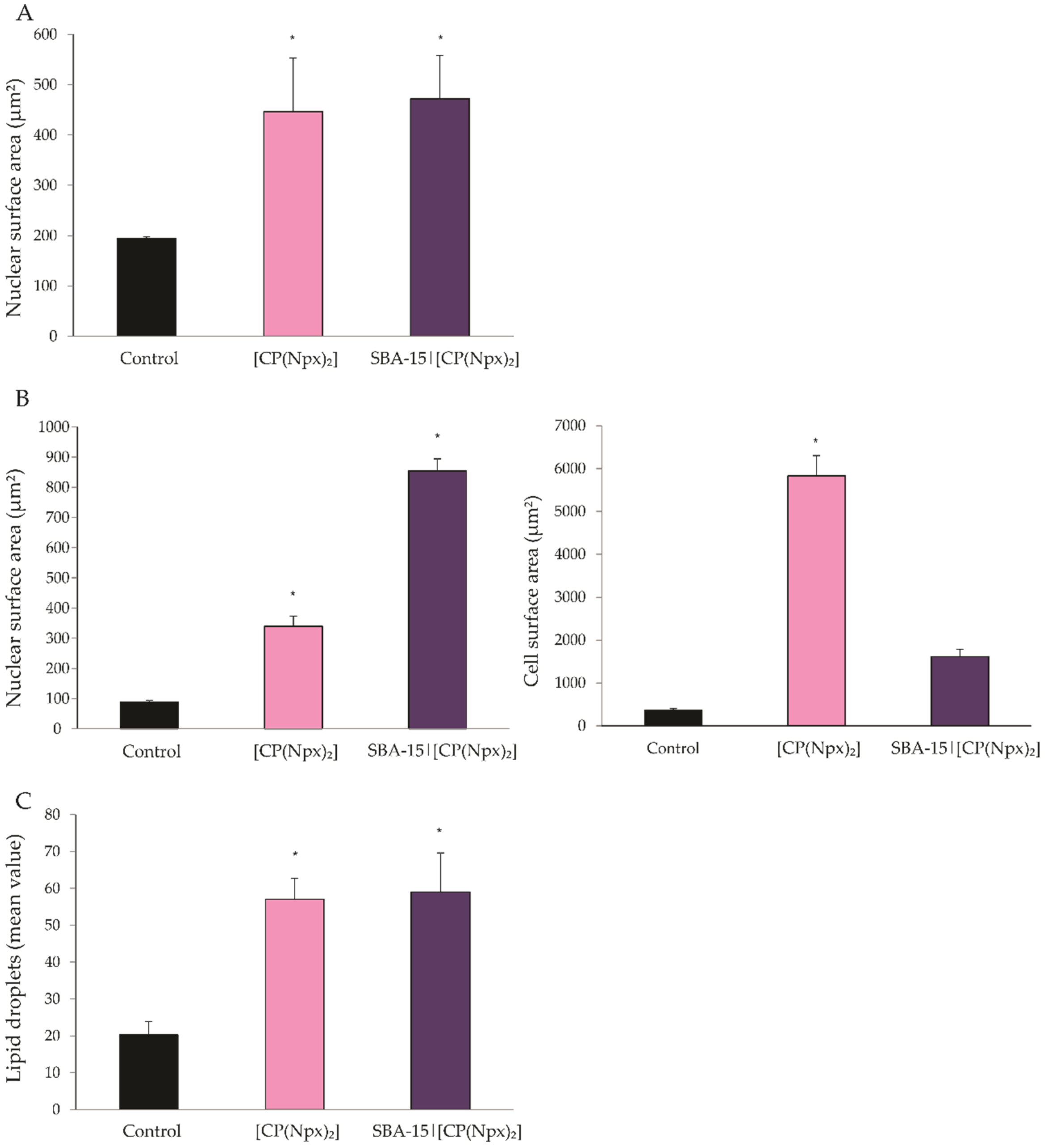

Figure S9: Effect of [CP(Npx)$_2$] and SBA-15|[CP(Npx)$_2$] on nuclear and cell size. B16 cells were treated with the IC$_{50}$ of [CP(Npx)$_2$] and the MC$_{50}$ dose of SBA-15|[CP(Npx)$_2$] for 72 h. Quantification of nuclear area after propidium iodide staining (A); nuclear area (left) and cell size (right) after hematoxilin-eosin staining (B); lipid droplets after Oil red O staining (C). * $p < 0.05$ compared to the untreated control.

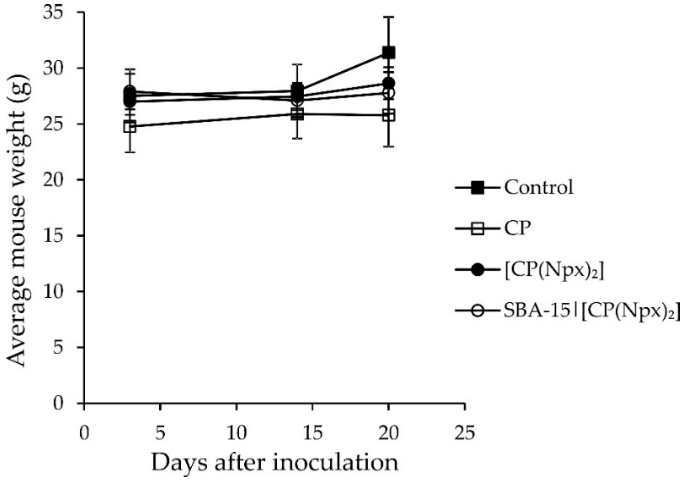

Figure S10: Average mouse body weight. Body masses of C57BL/6 mice were determined on days 3, 14 and 20 upon cell inoculation.

Table S1: Biochemical and hematological parameters of urine. Biochemical and hematological parameters of urine of C57BL/6 mice after the treatment with CP, [CP(Npx)$_2$] and SBA-15|[CP(Npx)$_2$]. Urine samples were collected on the day the animals were sacrificed.

Biochemical parameters	Control	CP	[CP(Npx)$_2$]	SBA-15|[CP(Npx)$_2$]
Leukocytes (leu/μL)	0 ± 0	0 ± 0	0 ± 0	0 ± 0
Nitrits	0 ± 0	1 ± 0	0.7 ± 0.6	1 ± 0
Urobilinogen (mg/dL)	2.6 ± 1.3	3.0 ± 1.0	3.3 ± 1.2	2.7 ± 2.2
Proteins (mg/dL)	54.7 ± 24.3	38.9 ± 25.0	68.8 ± 35.6	61.1 ± 6.7
pH	5.8 ± 0.5	6.0 ± 0	6.0 ± 0	5.9 ± 0.5
Blood (ery/μL)	0 ± 0	0 ± 0	0 ± 0	0 ± 0
Specific Gravity	1030 ± 0	1030 ± 0	1030 ± 0	1029.2 ± 1.4
Ketons (mg/dL)	0 ± 0	0 ± 0	0 ± 0	0 ± 0
Bilirubin (mg/dL)	0.2 ± 0.3	0.4 ± 0.3	0.3 ± 0.2	0.3 ± 0.2
